# Supplementary material for: Postpartum depressive symptoms following implementation of the 10 steps to successful breastfeeding program in Kinshasa, Democratic Republic of Congo: A cohort study
Source: PLoS Med. 2021 Jan 11;18(1):e1003465. doi: 10.1371/journal.pmed.1003465 (PMC7799755; doi:10.1371/journal.pmed.1003465)
Supplement: S1 Protocol — (DOCX) [file pmed.1003465.s001.docx]

**Title of Project:** Comparison of two clinic-based interventions to promote early initiation and exclusive breastfeeding through 6 months after birth in Kinshasa, Democratic Republic of Congo

**Country in which project will be conducted:** Democratic Republic of Congo (DRC)

**Full Name of Lead Organization:** University of North Carolina at Chapel Hill

**Name and Title of Project Director:** Marcel Yotebieng, MD, MPH, PhD.

Research Assistant Professor

UNC-DRC program

**Email:** [yotebieng@unc.edu](mailto:yotebieng@unc.edu)

Telephone: +243 81 827 1044

Fax: NA

Address: 4 Avenue Milambo,

Gombe, Kinshasa,

DR Congo

Duration of the project with anticipated start date: 24 months, anticipated start date – February 2011

Estimated total cost for the project ($US): 99,988

Amount requested from the A&T Small Grants Program: $99,988

**Summary of project** (< 200 words):

If at least 90% of children were exclusively breastfed for the first 6-months of life, the potential reduction in mortality that can be achieved will be higher than for any other interventions with sufficient evidence of effect. In the DRC >500,000 under-five deaths occurred in 2008. While >95% of children were breastfed at some point, 18% received something other than breast milk before initiation of breastfeeding, and half received something other than human milk by 1.4 months. Pre- and post-partum breastfeeding support has been shown to best improve the rate of exclusive breastfeeding (EBF). The global initiatives to improve breastfeeding practices have focused on maternity-level policies and procedures known as the Ten Steps to Successful Breastfeeding, which served as the basis for the Baby-Friendly Hospital Initiative. These quality of care steps impact hospital breastfeeding rates as well as breastfeeding rates throughout the 6 months postpartum period. However, EBF rates fall off rapidly in the DRC. In the proposed cluster randomized controlled trial, we plan to evaluate the effect of breastfeeding support provided by well-child clinic staff including the use of culturally appropriate messages in addition to the implementation of BFHI steps 1-9 Steps in maternities on the rate of breastfeeding initiation within 1 hour of birth and EBF throughout 6 months postpartum. If effective, this approach has great potential for scale up where most needed.

**B. Proposal Narrative (10 page limit)**

1. **Background and Rationale**

The global millennium development goal (MDG) # 4 targets a reduction by two thirds of the under-five mortality rate between 1990 and 2015. Globally, the total number of under-five deaths declined from 12.5 million in 1990 to 8.8 million in 2008 (1). However, progress has not been uniform. In 2003, a series of five scientific papers (2-6) known as “Child survival series” published in *The Lancet* drew considerable attention to the unacceptable high rates of child mortality that continue to burden low-income and poor areas of middle-income countries. To date, only 10 of the 67 countries with high child mortality rates (defined as 40 or more deaths per 1000 live births) are on track to meet the MDG target on child survival. All 34 countries with under-five mortality rates exceeding 100 per 1000 live births in 2008 are in sub-Saharan Africa, except Afghanistan. One in seven children in sub-Saharan Africa continue to die before their fifth birthday; the highest levels are observed in Western and Central Africa, where one in six children died before age five (169 deaths per 1,000 live births) (1). Virtually 1 in 5 child deaths that occurred worldwide in 2008 was in Nigeria or the Democratic Republic of Congo (DRC) (7). These high mortality rates are unacceptable as implementation of interventions for which there is sufficient (level 1) or limited evidence of effect (level 2) could prevent at least 63% of these deaths (4).

In 2008, another series (8-12) in *The Lancet* on maternal and child undernutrition highlighted the role of undernutrition as a preventable cause in over 3.5 million child deaths per annum. In the second paper in the series, Victora et al. showed in a pooled analysis of data from five long-standing prospective cohort studies from Brazil, Guatemala, India, the Philippines, and South Africa coupled with a systematic review of literature from developing countries, that poor fetal growth or stunting in the first two years of life leads to irreversible damage, including shorter adult height, lower attained schooling, reduced adult income, and decreased offspring birth weight (9). In addition, their findings indicate that children who are undernourished in the first 2 years of life and who put on weight rapidly later in childhood and in adolescence are at high risk of chronic diseases related to nutrition (9). The period from pregnancy to 24 months of age was highlighted as a crucial window of opportunity for reducing undernutrition and its adverse effects (11).

Human milk is the ultimate food to promote healthy growth and development of children under the age of two (13). It is recommended that infants be exclusively breastfed for the first six months of live and thereafter, to meet their evolving nutritional requirements, they should receive nutritionally adequate and safe complementary foods while breastfeeding continues for up to two years of age or beyond (14). Optimal breastfeeding practices, including initiation of skin to skin with breastfeeding within 1 hour of birth (controlling hypothermia), exclusive breastfeeding (EBF) with no additional fluid or food for 6 months, and complementing breastfeeding with age appropriate food after 6 months and continuation of breastfeeding until 24 months and beyond (15), have the potential to prevent 1.4 million child deaths annually (8). It has been estimated that sixteen percent of neonatal deaths could be averted if all infants were breastfed from day 1 and 22% if breastfeeding started within the first hour (16). If at least 90% of children were exclusively breastfed for the first 6-months of life, the potential reduction in mortality that can be achieved will be higher than for any other level-1 intervention (4). Yet, the current rates of EBF are far below 90% in most countries. In an analysis of recent national survey data, Black et al. reported that in Africa, Asia, and Latin America and the Caribbean only 25–31% of children 2–5 months of age were exclusively breastfed (8). Analyses by UNICEF of data from 111 countries between 1996 and 2004 showed considerable variation of EBF rates across regions: the highest rates being in East Asia/Pacific (43%) and Eastern/Southern Africa (41%), and the lowest in West/Central Africa (20%) (17).

With more than half a million child deaths in 2008, DR Congo bears the third largest burden of child deaths worldwide (7). These deaths are the result of a web of complex determinants (18), yet results from the 2007 DHS in DRC provide enough evidence to believe that breastfeeding practices play a major role in the extremely high infant mortality rate in the country. First, of the 9.2% of infants who die before the age of one, 4.2% die during the neo-natal period and the remaining 5% between 1 and 12 months. More deaths in the post-natal period suggest that factors behind these deaths are to be found among other sources in the feeding practices. Second, DHS data also show that by the age of 6 months, more than 10% of children in DRC are already stunted, virtually 15% already underweight-for-age and approximately the same percentage emaciated. Third, though breastfeeding is nearly universally accepted (95% of children were breastfed at some point), only 48% of infants studied in the 2007 DHS were initiated on breastfeeding within 1 hour of birth, 18% received something other than breast milk before initiation of breastfeeding, and half received something other than breastfeeding at 1.4 months (19). Much of the supplementation that occurs in western Africa is water or traditional foods, and may be amenable to change if change is supported by a trusted advisor. Therefore these data have to be understood in the context of relatively high utilization of primary health care. In fact, despite the challenges to accessing health care in DR Congo, DHS data showed that 85% of pregnant women attended at least one antenatal visit, 70% of live births occurred in a health facility (97% in Kinshasa). Of children 12 to 23 months of age, 71%, 59%, 45% received the first, second and third doses of DPT immunization administered according to the WHO immunization schedule (20) and the DRC’s Expanded Program of Immunization at 6, 10, and 14 weeks, while 63% had been immunized against measles (at 9 months). In this context, interventions to promote and support early initiation and continued exclusive breastfeeding through 6 months integrated into the primary health care delivery system, particularly when bundled with well-child visits have the potential to substantially impact child undernutrion and its adverse consequences.

The global initiatives to improve breastfeeding practices have focused on maternity-level policies and practices known as the *Ten Steps* which serve as the basis for the *Baby-friendly Hospital Initiative* (BFHI) (21). BFHI has recently been revised and updated to address intertwined HIV/AIDS issues (22). Originally launched in 1991 by the WHO and UNICEF following the Innocenti Declaration of 1990 (23), BFHI aims at improving the infant feeding guidance for pregnant women, mothers and newborns at health facilities that provide maternity services with the goal of protecting, promoting and supporting breastfeeding. BFHI, within its step 6, also supports, the International Code of Marketing of Breastmilk Substitutes (24). A maternity facility can be designated 'baby-friendly' when it has implemented the *Ten Steps* to support successful breastfeeding including (1) having a written breastfeeding policy that is routinely communicated to all health care staff, (2) training all health care staff in skills necessary to implement this policy, (3) informing all pregnant women about the benefits and management of breastfeeding, (4) helping mothers initiate breastfeeding within one half-hour of birth, (5) showing mothers how to breastfeed and maintain lactation, even if they should be separated from their infants, (6) giving newborn infants no food or drink other than breast milk, unless medically indicated and when it does not accept free or low-cost breastmilk substitutes, feeding bottles or teats, (7) allowing mothers and infants to remain together 24 hours a day, (8) encouraging breastfeeding on demand, (9) giving no artificial teats or pacifiers to breastfeeding infants, and (10) fostering the establishment of breastfeeding support groups and refer mothers to them on discharge from the hospital or clinic.

Although implementation of the Ten Steps has been shown to improve the rate of EBF (10,25), its effect may dampen after discharge from maternities unless Step 10 is well implemented – establishing breastfeeding support in the community. In a cluster randomized controlled trial (RCT) in Belorussia, in which 31 maternity hospitals and polyclinics were randomized to receive an intervention based on the Ten Steps (n=16) vs. usual care (n=15), the authors reported that the proportion of children initially breastfed at baseline was 100% in both groups. However, infants from intervention sites were significantly more likely than infants from control sites to be breastfed to any degree at 12 months (19.7% vs. 11.4%; adjusted odds ratio, 0.47; 95% confidence interval, 0.32-0.69), and to be exclusively breastfed at 3 months (43.3% vs. 6.4%; P<.001) and at 6 months (7.9% vs. 0.6%; P =.01). In another RCT conducted among low-income women in Brazil evaluating the effect of adding a strong Step 10 with postnatal home visits to BFHI (steps 4 – 9) vs. BFHI (steps 4 – 9) alone, the authors reported that the proportion of EBF decreased from 70% at hospital discharge in both groups to 45% and 25% in the intervention group at 3 and 6 months compared to 10% and 4% respectively, in the control group (26), suggesting that addition of post-natal support can improve the duration of breastfeeding even in areas where breastfeeding acceptance is low. In fact, evidence from two recent independent meta-analyses (27,28), show that though provision of any support at any time (pre- or post-natal) improved breastfeeding practices, combinations of pre- and post-natal interventions had the strongest effect. However, as studies included in these meta-analyses demonstrate, virtually all post-natal interventions to support breastfeeding have been community-based despite the fact that following discharge from maternities, the mother-infant pair continues to have frequent contacts with primary health care providers.

Mothers and caregivers are advised to bring their healthy children to the primary health care clinic generally referred to as “well-child” or “well-baby” clinic for regular check-ups. The American Academy of Pediatrics recommends up to 21 visits before the age of 5 years (29). DRC follows the WHO’s 1998 model of care, which suggest postnatal visits within six hours after birth, three to six days, six weeks and six months (6-6-6-6) (30). The six days visit usually occurred 1 week after discharge from maternity. Starting at 6 weeks, , the well-child clinic visits are generally every 4 weeks until the baby is 6 months and every 3 months thereafter until the baby turns 24 months when visits continue on yearly basis. During these visits, the child is examined, growth parameters are measured and recorded on the “road to health” card, and the child is immunized according to the expanded immunization program schedule. Although adherence to well-child clinic visits in DRC is not well documented, the relatively good immunization coverage, suggests at least reasonable attendance. These regular contacts of the mother-infant pair with professional health care providers represent an ideal opportunity for health education, including reinforcement of messages and promotion of EBF.

Most mothers stop exclusively breastfeeding because of the perception that their infant is not getting enough or is not satisfied with breast milk alone (31). Our hypothesis is that empowered with more knowledge and skills, health care staff in well-child clinics can successfully counsel these mothers to continue EBF and those who are having difficulties breastfeeding on how to manage these difficulties.

In this study, we propose to implement the innovative idea of using well-child clinic staff to provide breastfeeding support using culturally appropriate educational messages including metaphors in combination with the implementation of BFHI steps 1 through 9 in maternities, and evaluate their impact on the rate of early initiation of breastfeeding and duration of exclusive breastfeeding during the first 6 months.

1. **Objectives**

This study has two primary objectives

1. Estimate the effect of implementing BFHI steps 1-9 in maternities on the rate of breastfeeding initiation within 1 hour of birth and on the rate of exclusive breastfeeding at 6 months
2. Estimate the effect of the innovative idea of providing breastfeeding support including the use of culturally appropriate educational messages and metaphors in well-child clinics as the “community’ basis of Step 10, in combination with the implementation of BFHI steps 1-9 in maternities on the rate of exclusive breastfeeding at 6 months
3. **Project Design and Implementation Plan**

This will be a randomized controlled trial using cluster randomization. The unit of randomization will be health facilities (cluster) as the experimental or control interventions will be delivered at the level of maternity and well-child clinic of each participating health facility. The study will be conducted in Kinshasa, the capital of the DRC. BFHI is not being implemented to any extent today in DRC. The main attempt to implement BFHI steps in DRC was let by UNICEF in early 2000s as part of a national campaign of breastfeeding promotion, Overall 25 out of more than 6,000 health facilities eligible were certified through this effort including 13 in Kinshasa. The last hospital certified was in 2004 in the Katanga province when the funding stopped. Just two years after the peace deal that ended the deadliest war since World War II, which, in addition to decades of gross mismanagement and rampant corruption, have left the country infrastructures in shambles (32,33), DRC was simply not ready to take over the initial UNICEF efforts. Currently, pregnant women attending antenatal clinic (ANC) as well as nursing mothers attending well-child clinic visits are given some information about the benefits and management of breastfeeding. But there are no quality of care steps being implemented and where it exist, the supporting materials to carry out effective counseling of mothers date from the UNICEF supported campaign 10 years ago.

Since 2002 and under the leadership of Dr Behets, the University of North Carolina at Chapel Hill (UNC) has collaborated with the Congolese Ministry of Health (MoH) and national and local organizations to improve Human Immunodeficiency Virus (HIV) prevention and care through the University Technical Assistance Program (UTAP) with support from various donors. Currently, 44 maternities are supported by our UNC-DRC team serving more than 50,000 pregnant women yearly. These maternities are generally part of a health facility that also hosts a well-child clinic where infants are typically monitored on a monthly basis. As stated above, routine immunization takes place during these monthly visits. To minimize the implementation cost, six of the 44 health facilities with the largest number of deliveries per year will be selected to be part of the study – 3 in urban and 3 in peri-urban areas. Prior to selection, direct observation will be carried out in eligible health facilities to collect information on key characteristics (workload per staff, type of management [government vs private], general infrastructure) that are likely to affect the quality of care. Facilities will be matched for these characteristics prior to randomization. This approach will ensure for example, that health care facilities at the periphery of the city that might have lower patient volumes than facilities in urban areas and serve populations more likely to be socio-economically disadvantaged are included. The selected six facilities will be randomized into 3 groups to receive the following interventions: 1) continuation of current care practices (usual care), 2) implementation of BFHI steps 1-9 in maternities (BFHI steps 1-9), and 3) implementation of BFHI steps 1-9 in maternities and provision of breastfeeding support including culturally appropriate educational messages and metaphors as the ongoing aspect of step 10 in well-child clinic (BFHI steps 1-9 +well-child clinic). The study will be implemented in 2 phases.

Pre-intervention phase

This phase itself can be sub-divided in three sub-phases: an assessment phase, a hospital readiness phase, and a messages development phase. Direct observations of maternities and well-child clinics in the selected health facilities will be conducted to assess environmental and other barriers to early infant feeding. Health professionals including doctors, paramedical and support staff in each of the clinics will be interviewed with a structured questionnaire to assess their knowledge, attitudes, and practices regarding breastfeeding support in their facilities. Semi-structured key informant interviews with the head of each health facility, key stakeholders from the breastfeeding promotion program from the MoH, mother-infant pairs attending purposively-selected maternities and well-baby clinics (4-5 per health facility; total: 25-30 interviews) will be conducted with study staff under the supervision of Pr Lapika, a social scientist and Chair of the Department of Anthropology at the University of Kinshasa with a strong expertise in qualitative methods and data analyses. This process will be informed by research on-going at UNC’s Carolina Global Breastfeeding Institute on “Organizational readiness to change” on the Ten Steps. Information collected from this initial assessment will be used by a technical working group of key researchers and public health practitioners including Pr Lapika as well as community members to develop culturally appropriate messages including metaphors designed to support exclusive breastfeeding and address the challenges and opportunities identified in the assessment sub-phase. These key messages will be included in posters for well-child clinics and educational brochures in French and Lingala (the most commonly used languages in Kinshasa) to be distributed to mothers at the well-child clinic visits. During this Phase we will evaluate how to best adapt the *Ten Steps* of the BFHI to the local context. Dr Labbok, international expert in breastfeeding, and Dr Behets, who has 20+ years of public health experience in sub-Saharan Africa, will provide technical advice throughout all phases of the proposed project.

Intervention Phase

Following randomization of health facilities to one of the three interventions - BFHI steps 1-9, BFHI steps 1-9 plus well baby, usual care - each hospital in the BFHI steps 1-9 and BFHI steps 1-9 plus well baby groups will be assessed as to readiness for implementing the *Ten Steps* and the hospital leadership – medical and administrative - will be provided with evidence on the importance of the *Ten Steps*. Health professionals (including doctors and nurses) and support staff of maternities randomized to BFHI steps 1-9 and maternities and well-child clinics randomized to - BFHI steps 1-9+well child clinic arms will be provided with 20 hours of training with the UNICEF/WHO course (34). In addition, staff of maternities from facilities randomized to BFHI steps 1-9 and those from maternities and well-child clinics randomized to BFHI steps 1-9+well child clinic arms will receive two additional hours of training on how to listen, to learn from mothers, to establish good relationships, to build mothers' confidence, and to offer support, taken from the WHO/UNICEF Breastfeeding Counseling Course (35). The training will be done in groups with actual mothers and babies in well-child clinics followed by a 30-minute debriefing in groups for the staff to share their experiences and give feedback. Copies of the UNICEF norms and existing guidelines for the encouragement of breastfeeding (36) will be offered to all health facility managers randomized to BFHI steps 1-9 and BFHI steps 1-9+well child clinic arms and support will be provided, as needed, for the implementation of each of the *Ten Steps*. In DRC, following discharge from maternity, mother and infant are expected to return to the delivery facility for birth certificate and a general check-up of both the mother and her infant. But this visit is often neglected by maternities and attendance is generally low. In maternities randomized to interventions, an effort will be made to make sure that mother and baby effectively return for this visit including home visit for those who fail to return in the first 3 weeks. To maintain a continuous contact and ensure that mothers who need support can access it during the period between the first week visit in the maternity and the first well-child clinic visit at 6 weeks, the phone number of the maternity nurse will be given to each mother and she will be encouraged to call or return to the maternity if she experience any difficulty breastfeeding or has any question during this period. An effort will be made to assist clinic staff in handling telephone calls through the development of a job aid for handling common problems, modified from the UNC clinical job aids.

It is recognized that this short-cut (without active multi-level task force development and without other aspects of the BFHI [credentialing]) approach may not result in full implementation, so an assessment of the progress, status, and continued adherence to the *BFHI Steps* will take place throughout the intervention phase. Posters with key exclusive breastfeeding promotion messages will be posted and mothers in these facilities will also receive educational brochures in French and Lingala at the well-child clinic visits. Data from the 2007 DSH show that 85% of people in Kinshasa have more that secondary education, and more than 91% of the population is literate. All these supports will only be provided in facilities randomized to intervention groups. Health professional and staff of facilities randomized to usual care arm will receive no training and no documentation.

**Figure 1.** Scheme of the study design

Development of culturally appropriate messages and educational materials

Initial Assessment

(O)

(

R

BFHI steps 1-9 alone

BFHI steps 1-9 + well-baby clinic

Intervention Evaluation

Intervention Evaluation

Usual care

Intervention Evaluation

1. **Evaluation**

The goals of this project are to evaluate a) the effect of this short-cut approach to implementation of BFHI steps 1-9 in maternities in Kinshasa on the rates of early initiation of breastfeeding and that of EBF at 6 months, compared to the usual care arm and b) the effect of breastfeeding support provided in well-child clinics in addition to the short-cut implementation (without accreditation) of BFHI steps 1-9 in maternities on the rate of EBF 6 months post-partum, compared to the BFHI steps 1-9 arm. WHO definitions will be used (37):[7](http://www.sciencedirect.com.libproxy.lib.unc.edu/science?_ob=ArticleURL&_udi=B6T1B-4H5GDN3-W&_user=130907&_coverDate=09%2F30%2F2005&_rdoc=1&_fmt=high&_orig=search&_origin=search&_sort=d&_docanchor=&view=c&_acct=C000004198&_version=1&_urlVersion=0&_userid=130907&md5=7b6d09c61c88f94f9356fb50cc5be97d&searchtype=a#bbib7) WHO, *Indicators for assessing breastfeeding practices*, WHO, Geneva (1992). infants will be classified as exclusively breastfed if they received only breast milk (no water, other liquids, or solids) and as early initiation of breastfeeding if breastfeeding is initiated within one hour after birth. Secondary outcomes including any breastfeeding and predominant breastfeeding will also be considered. Information on breastfeeding practices will be collected via questionnaires administered to mothers at maternity discharge, first weeks visit, and at every well-child visit up to 6 months post-partum. Although masking of the interventions will not be possible, independent interviewers will be appropriately trained and questionnaires designed in a way to help minimize reporting bias (using scripts for example to de-stigmatize undesirable answers).

The extent to which we can attribute outcomes to interventions depends heavily on the success of the implementation of the 2 interventions (short-cut BFHI steps 1-9 and well-baby intervention) and these are included in the evaluation design. Step 1 is easy to document. For Step two, a pre- and post-training test will be used to assess the improvement in the skill and knowledge of the trained health staffs. A section in the questionnaire that will be used to interview mothers and collect information on breastfeeding will focus on documenting their exposure to activities related to the implementation of Steps 3 to 9 in the delivery facilities and activities related to the implementation of modified steps in well-baby clinics. Step 10 will include provision of the brochure and orientation to attending the well-baby clinics.

This proposed study is a cluster randomized controlled trial of interventions designed to support breastfeeding in maternities and well-child clinics in Kinshasa. Following randomization and 1 month post training completion (to allow staff in intervention groups to gain some experience practicing their newly acquired skills on breastfeeding counseling and support and to gain support in the implementation of all 10 steps), recruitment will start. A cohort of 900 consecutive mothers of healthy infants being discharged from participating maternities (150 per maternity), who intend to attend well-baby clinic visits in the same health care facilities until the child will be at least 6 months, will be recruited and followed-up until the child will be 6 months old. Mothers will be asked to return for the first week post-discharge visit in the maternity and for all other follow-up visits in the well-child clinic on a monthly basis starting at 6 weeks post-partum to match the routine well-child clinic visit schedule. As stated above, they will also be encouraged to return to clinic or call their care provider if they experience any difficulty with breastfeeding. Permission will be obtained from participating mothers to contact them by phone or visit them at home in case they might miss an appointment. Structured interviews will be administered to participating mother-infant pairs at enrollment (baseline), at 1, 6, 10, 14, 18, and 24 weeks. In addition to socio-demographic characteristics of the mother-infant pair that will be collected at baseline, during these interviews information on the maternity experience, the well-baby experience, and on infant feeding practices will be collected and mothers will be asked whether their infant experienced any severe diarrhea or any sign or symptom of lower respiratory track infection since the last interview, and if so, to describe the episodes and whether they took the infant to a health care provider.

Data from the questionnaires will be double entered into an Access database, compared for consistency and cleaned as needed. The proportion of infants initiating breastfeeding within 1 hour of birth and who are exclusively breastfed at 14 weeks and 6 months post-partum are the primary outcomes that will be measured and compared between intervention and control groups. To estimate the effect of implementing the *Ten Steps* in maternities on the rate of breastfeeding initiation within 1 hour of birth and that of exclusive breastfeeding at 6 months, the usual care group will provide the counterfactual, and the proportion of infants with each of the primary outcomes in the BFHI steps 1-9 group will be compared to the proportion of infants with the same outcomes in the usual care group. To determine the effect of providing breastfeeding support in well-child clinics in addition to implementing the Steps 1-9 in maternities on the rate of EBF at 6 months, the BFHI steps 1-9 group will serve as control and the proportion of infants with the outcome in the intervention group ( BFHI steps 1-9+well-child clinic) will be compared to that of infants with the outcome in the BFHI steps 1-9 arm.

All analyses will be conducted by intent-to-treat (38), meaning that mother-infant couples will be classified to have received the intervention that their facility was randomized to whether they continued to come to the facility or not after initial discharge from the maternity and the level of uptake of the *Ten Steps* in the facility. Baseline characteristics of mother-infant pairs will be compared between interventions and control groups to check for any imbalance that may persist despite randomization. Pearson chi-square will be used to compare categorical variables and T-test will be used for continuous variables assuming they are normally distributed. Normality will be formally evaluated using Kolmogorov–Smirnov test. For non-normally distributed continuous variables, Wilcoxon Rank-sum test will be used. Generalized Estimation Equation (GEE) and Log Binomial model will be used to estimate the prevalence ratio comparing interventions groups to controls. In GEE, the dependence within cluster is treated as nuisance, and valid inference (or confidence interval) is produced for population average effects as long as the mean structure is correctly specified, even if the dependence structure is misspecified (39).

The sample size of 150 mother-infant pairs per maternity is based on a series of power calculations using various prevalence estimates of the prevalence of EBF at 6 months in the control group (15% to 35% for usual care) and a minimum expected effect size of RR = 1.5 for the first objective – comparison of BFHI steps 1-9 to usual care (figure 2.a) and a prevalence of EBF at 6 months in the BFHI steps 1-9 group of 45 to 65% and a minimum expected effect size of RR = 1.3 for the second objective – comparison of the provision of support in the well-child clinics in addition to the implementation of BFHI steps 1-9 in maternities vs. BFHI steps 1-9 in maternities alone (figure 2.b). Assuming two-tailed Person chi-square test with α values of .05, the sample size of 900 mother-infant pairs should provide adequate power to detect a modest difference in the proportion of infants exclusively breastfed at 6 months between BFHI alone and usual care arms or between BFHI steps 1-9 + well-child clinics arm and BFHI steps 1-9 alone.

To determine the number of clusters of size n = 150 that will be needed to achieve an 80% power, we used the formula proposed by Hayes and Bennett (40). Assuming a coefficient of variation of 25% (which is generally considered reasonable), and a 20% prevalence of exclusive breastfeeding in the “usual care” group, we varied various expected prevalence of EBV that could be achieve with the implementation of the Ten Steps. Results are presented in the figure 3 below. Once the prevalence of EBF in the intervention group reaches 40%, the number of clusters needed falls below 4.

**Figure 2.** Power estimate curves for: a- detecting a minimum effect size of RR = 1.5 comparing the prevalence of EBF at 6 months in the BFHI steps 1-9 group to that in the usual care group and b- detecting a minimum effect size of 1.3 comparing prevalence of EBF at 6 months in BFHI steps 1-9 + well-child clinic group to that in BFHI steps 1-9 group for a sample of n = 150 per health facility, n = 300 per randomization group, and n = 900 total for various prevalences of exclusive breastfeeding at 6 months.

Figure 3. Number of clusters of size 150 needed to achieve 80% power for a fixed coefficient of variation of 0.25 by the expected proportion of EBF in the intervention group, assuming a 20% prevalence of EBF in the usual care group.

Assuming a loss to follow-up rate of 10% over the 6 months - the duration of the follow-up in the study, the effective sample size that will be available for analysis is 135. With this effective sample size, our study will have an 88% power to detect a modest 30% increase in the prevalence of exclusive breastfeeding at 6 months in the intervention group – corresponding to a change as small as an increase from the current proportion of EBF at 6 months of 17% (assuming it stays the same in the usual care group) to 22.5% after implementing BFHI steps 1 through 9.

At the end of the evaluation, the findings of the study will be shared with all facilities that took part in the study and with the community. Facilities in the usual care arm will be encouraged to implement the most successful intervention.

1. **Sustainability and Potential for Scaling-Up**

As described in the background section, the major problem for breastfeeding in DRC and to some extent in Western/Central Africa is not its acceptability, but the continuation of exclusive breastfeeding. The innovative idea we propose to evaluate is whether provision of breastfeeding support including educational messages by health care professionals during well-child clinic visits, in combination with initial support through the *Ten Steps* in the maternity, will result in improved duration of exclusive breastfeeding. The advantage of well-child clinics is that they are used for prophylaxis visits. Mothers may be less worried during these visits as compared to during pregnancy or immediately after delivery or when the visit is motivated by an illness. They might also be less distracted compared to when the visit is at home and probably open to listening to advice, particularly if given by a supportive and skilled health professional. Moreover, these visits start happening early enough (6 weeks) that there are ample opportunities to correct any sub-optimal practice that may have started between the discharge from maternity and the first visit. In DRC and in most sub-Saharan Africa including Western/Central Africa in particular, the average rate of immunization, a proxy for the attendance rate of well-child clinic visits, is relatively good. If we manage to show that provision of extra support for breastfeeding in well-child clinics significantly results in higher proportion of infants exclusively breastfed at 6 months, this type of intervention has great potential for massive scale-up in DRC and Africa where the needs are especially great, but even in the developed world were attendance to well-baby clinic visits is even higher.

Our UNC-DRC team works collaboratively with the Ministry of Health and other non-governmental organizations. The national program on nutrition best known by its French acronym – PRONANUT – is the technical program of the MoH involved in the active promotion of breastfeeding nationwide. They will be implicated in this study from the design of educational messages to the organization of the training for health professional in selected health facilities. If we are successful in showing that additional support provided during well-child visits can help improve the rate of exclusive breastfeeding, we will encourage scale-up the intervention in the country. Evaluating the effect of a rigorous implementation of BFHI steps with necessary local adaptation in maternities has never been done in DRC and will allow the PRONANUT to retool their breastfeeding promotion efforts effort as well.

1. **Organizational Capacity and Key Personnel**

Our study team (UNC-DRC) has an established history of collaborative work in Kinshasa. In 2003, with funding from the CDC Global AIDS Program (GAP), a research team from the University of North Carolina led by Dr. Behets began collaborating with the Congolese MoH and national and local organizations to improve Human Immunodeficiency Virus (HIV) prevention and care through the University Technical Assistance Program (UTAP) and with support from several other donors. In 2008, this work continued under a new agreement with the CDC entitled “PACT: Providing AIDS Care and Treatment in the Democratic Republic of Congo under the President’s Emergency Plan for AIDS Relief.” These activities are conducted in partnership with the Kinshasa School of Public Health (KSPH) and are designed to support HIV prevention, treatment and care programs for children and adults, including patients co-infected with tuberculosis (TB) and HIV and prevention of mother-to-child transmission (PMTCT). Additional support was obtained from the Global Fund to Fight AIDS, TB and Malaria, UNICEF, the Clinton Foundation, and the Elizabeth Glaser Pediatric AIDS Foundation. The UNC/KSPH partnership has succeeded in establishing a network of HIV service delivering facilities which currently consists of 44 maternities providing PMTCT services, 20 TB clinics providing HIV voluntary testing and counseling (VCT), one primary health center, and one pediatric hospital providing family-centered comprehensive HIV/ Acquired Immune Deficiency Syndrome (AIDS) care and treatment services. In 2008, the UNC-DRC team received funding from the Elizabeth Glaser Pediatric AIDS Foundation to conduct operations research that describes where and how antenatal and delivery services are provided in Kinshasa outside the main health care facilities in order to identify and test strategies for further PMTCT roll-out.

A key partner in the implementation of our projects has been the Kinshasa School of Public Health (KSPH) with whom UNC has worked since 2000. Other partners include the Bureau *des Oeuvres Médicales Diocésaines* (Catholic Health Board) the Salvation Army, MOH agencies such as the National AIDS Control Program (PNLS), the National Tuberculosis Control Program (PNT), and PRONANUT as well as international organizations including the Elizabeth Glaser Pediatric AIDS Foundation, UNICEF, Médecins Sans Frontières, Action Against Hunger, the German Agency for Technical Cooperation (GTZ), the Clinton Foundation and the Global Fund to Fight AIDS, Tuberculosis, and Malaria.

In collaboration with the KSPH, we have established technical, administrative/logistical and financial support systems based at a central office in Kinshasa. Our proposed management and implementation plan builds on this collaboration, and takes advantage of the extensive experience and structures already in place.

**Roles of Contributing Partners**

The prime partner, UNC-CH will serve to provide overall financial and human subjects supervision. The Project Director or Principal Investigator (PI), Dr. Marcel Yotebieng, MD, MPH, PhD, is Assistant Professor of Epidemiology at the UNC Gillings School of Global Public Health and co-investigator on the CDC cooperative agreement – PACT. He is based in Kinshasa where he provides technical advice regarding the HIV-related activities. Dr Yotebieng will be responsible for overall project planning, implementation and evaluation. UNC will be responsible for the financial distribution of the grant award to the sub-recipient as well as for the financial and program reporting to Alive & Thrive. UNC-CH will ensure adherence to all applicable Alive & Thrive and USG regulations. All protocols, data collection instruments and participant informed consent forms will require the approval of both the UNC-CH and KSPH Institutional Review Boards before implementation, Drs. Frieda Behets, PhD, MPH, Professor of Epidemiology at UNC-CH and lead investigator in the UNC-DRC program and Miriam Labbok, MD, MPH, FACPM, IBCLC, Professor of Maternal and Child Health and Director of Carolina Global Breastfeeding Institute at UNC-CH, will provide assistance in the design and oversight of the conduct of this study. Dr. Behets has more than 20 years of public health experience in sub-Saharan Africa and is the principal investigator of PACT. Dr Labbok, an international expert on breastfeeding, has more than 35 years of research and program work on maternal/child dyad health and nutrition. She is known for the development of the Lactational Amenorrhea Method (LAM) for birth spacing, technical secretariat for the Innocenti Declaration meetings, definitions for breastfeeding, studies of health consequences of breastfeeding for the dyad; and for community health initiatives using operational and translational research approaches. She has served previously as Senior Advisor for Infant and Young Child Feeding and Care at UNICEF where she oversaw the revision and updating of the BFHI materials that now include the expanded concept of support in well-baby clinics. She currently is the PI and clinical advisor in projects introducing the Ten Steps in nearly 30 hospitals. Dr. Lapika, Chair of the Department of Anthropology at the University of Kinshasa and Director of CERDAS **(***Centre for the Coordination of Social Science Research and Documentation in Africa South of the Sahara),* has strong expertise in qualitative methods and data analyses and more than 2 decades of experience in social research in DRC. He has worked with Drs. Behets and Yotebieng on the PMTCT project and supervises a team of research assistants in Kinsahsa., He will provide scientific support, and will ensure cultural appropriateness of strategies and planned educational materials. He will support the In-Country Project Director to implement the activities described in the technical approach including active participation in the planning, conduct and analysis of the semi-structure discussion groups supervision visits to implementation sites.

Local field implementation of activities will be the responsibility of KSPH in collaboration with the UNC-CH In-Country Project Director. In collaboration with the In-Country Project Director, KSPH will oversee timely implementation of field activities and ensure that all activities and results requirements are met. The KSPH will be responsible for: hiring, administrative management and institutional oversight of the local hires; all local logistics support related to project activities, office operations, and local financial management; reporting of any adverse events or human subjects violations regarding IRB approved protocols; and data collection and participant interaction.

Other project contributors include non-governmental organizations such as the Armée du Salut (AS) and Bureau Diocésain des Œuvres Médicales de Kinshasa (BDOM). UNC/KSPH will select six health facilities from a network of forty-four within the DRC for the proposed study. Most of these facilities if not government owned are part of either BDOM or AS health network. AS and BDOM will also be keys partners in the dissemination and scale-up of the findings from this study. Our key partners have agreed to provide staffing and other assistance toward local field implementation on this research initiative. The Congolese MOH will act in an advisory capacity offering assistance as needed.

The KSPH/UNC-DRC partnership functions based on contractual agreements and a manual of procedures that describes the management policies and procedures regarding personnel, communications and lines of authority, and collaboration with other partners and programs. The Director of the KSPH has overall responsibility for adherence to in-country regulations, labor code, as well as USG regulations. The Director of the KSPH has also overall responsibility for monitoring, evaluation and financial management.

**C. Timeline (1 page limit)**

**D. References (4 page limit)**

(1) United Nations. The Millennium Development Goal - Report 2010. 2010; Available at: <http://www.un.org/millenniumgoals/pdf/MDG%20Report%202010%20En%20r15%20-low%20res%2020100615%20-.pdf>. Accessed November 23, 2010.

(2) Black RE, Morris SS, Bryce J. Where and why are 10 million children dying every year? The Lancet 2003 6/28;361(9376):2226-2234.

(3) Bryce J, el Arifeen S, Pariyo G, Lanata CF, Gwatkin D, Habicht J. Reducing child mortality: can public health deliver? The Lancet 2003 7/12;362(9378):159-164.

(4) Jones G, Steketee RW, Black RE, Bhutta ZA, Morris SS. How many child deaths can we prevent this year? The Lancet 2003 7/5;362(9377):65-71.

(5) Victora CG, Wagstaff A, Schellenberg JA, Gwatkin D, Claeson M, Habicht J. Applying an equity lens to child health and mortality: more of the same is not enough. The Lancet 2003 7/19;362(9379):233-241.

(6) Claeson M, Gillespie D, Mshinda H, Troedsson H, Victora CG, Bellagio Study Group on Child Survival. Knowledge into action for child survival. Lancet 2003 Jul 26;362(9380):323-327.

(7) Black RE, Cousens S, Johnson HL, Lawn JE, Rudan I, Bassani DG, et al. Global, regional, and national causes of child mortality in 2008: a systematic analysis. The Lancet 2010 6/5;375(9730):1969-1987.

(8) Black RE, Allen LH, Bhutta ZA, Caulfield LE, de Onis M, Ezzati M, et al. Maternal and child undernutrition: global and regional exposures and health consequences. The Lancet 2008 1/19;371(9608):243-260.

(9) Victora CG, Adair L, Fall C, Hallal PC, Martorell R, Richter L, et al. Maternal and child undernutrition: consequences for adult health and human capital. The Lancet 2008 1/26;371(9609):340-357.

(10) Bhutta ZA, Ahmed T, Black RE, Cousens S, Dewey K, Giugliani E, et al. What works? Interventions for maternal and child undernutrition and survival. The Lancet 2008 2/2;371(9610):417-440.

(11) Bryce J, Coitinho D, Darnton-Hill I, Pelletier D, Pinstrup-Andersen P. Maternal and child undernutrition: effective action at national level. The Lancet 2008 2/9;371(9611):510-526.

(12) Morris SS, Cogill B, Uauy R. Effective international action against undernutrition: why has it proven so difficult and what can be done to accelerate progress? The Lancet 2008 2/16;371(9612):608-621.

(13) WHO, UNCEF. Global strategy for infant and young child feeding. 2003; Available at: <http://whqlibdoc.who.int/publications/2003/9241562218.pdf>. Accessed November 23, 2010.

(14) Michael S. Kramer, Risuka Kakuma. Delayed breastfeeding initiation increases risk of neonatal mortality. Pediatrics 2006;117(3):e380-e381; e382, e383, e384, e385 e386.

(15) Kramer MS, Kakuma R. Optimal duration of exclusive breastfeeding. Cochrane Database Syst Rev 2002;(1)(1):CD003517.

(16) Edmond KM, Zandoh C, Quigley MA, Amenga-Etego S, Owusu-Agyei S, Kirkwood BR. Delayed breastfeeding initiation increases risk of neonatal mortality. Pediatrics 2006 Mar;117(3):e380-6.

(17) UNICEF. Progress for Children - Exclusive breastfeeding. Available at: <http://www.unicef.org/progressforchildren/2006n4/index_breastfeeding.html>. Accessed November 18, 2010.

(18) Mosley W, Chen L. *An analytical framework for the study of child survival in developing countries*. Popul Dev Rev 1984;10(Suppl):24-25.

(19) Ministère du Plan avec la collaboration du Ministère de la Santé Kinshasa, République Démocratique du Congo., Macro International Inc.
Enquête Démographique et de Santé. ; 2007.

(20) WHO. WHO recommendations for routine immunization - summary tables. 2010; Available at: <http://www.who.int/immunization/policy/immunization_tables/en/index.html>. Accessed November 29, 2010.

(21) Naylor AJ. Baby-Friendly Hospital Initiative. Protecting, promoting, and supporting breastfeeding in the twenty-first century. Pediatr Clin North Am 2001 Apr;48(2):475-483.

(22) WHO., UNICEF. Baby-friendly hospital initiative : revised., updated and expanded for integrated care. 2009; Available at: <http://www.unicef.org/nutrition/files/BFHI_2009_s3.1and2.pdf>. Accessed November 18, 2010.

(23) WHO, UNICEF. Innocenti declaration on infant and young child feeding. 2005; Available at: <http://www.innocenti15.net/declaration.pdf.pdf>. Accessed November 23, 2010.

(24) WHO. International Code of Marketing of Breastmilk Substitutes 1981; Available at: <http://www.who.int/nutrition/publications/infantfeeding/9241541601/en/index.html>. Accessed November 18, 2010.

(25) Abrahams SW, Labbok MH. Exploring the impact of the Baby-Friendly Hospital Initiative on trends in exclusive breastfeeding. Int Breastfeed J 2009 Oct 29;4:11.

(26) Coutinho SB, de Lira PIC, de Carvalho Lima M, Ashworth A. Comparison of the effect of two systems for the promotion of exclusive breastfeeding. The Lancet 2005 9/24;366(9491):1094-1100.

(27) Britton C, McCormick FM, Renfrew MJ, Wade A, King SE. Support for breastfeeding mothers. Cochrane Database Syst Rev 2007 Jan 24;(1)(1):CD001141.

(28) Chung M, Ip S, Yu W, Raman G, Trikalinos T, DeVine D, et al. Interventions in Primary Care to Promote Breastfeeding: A Systematic Review. Prepared for the Agency for Healthcare Research and Quality by the Tufts-New England Medical Center Evidence-based Practice Center, under Contract No. 290-02-0022. 2008 October;AHRQ Publication No. 08-05125-EF-1.

(29) Committee on Practice and Ambulatory Medicine. Recommendations for Preventive Pediatric Health Care. Pediatrics 2007 December;120(6):1376.

(30) WHO. Postpartum care of the mother and newborn: Report of a technical working group. 1998.

(31) Li R, Fein SB, Chen J, Grummer-Strawn LM. Why mothers stop breastfeeding: mothers' self-reported reasons for stopping during the first year. Pediatrics 2008 Oct;122 Suppl 2:S69-76.

(32) Coghlan B, Brennan RJ, Ngoy P, Dofara D, Otto B, Clements M, et al. Mortality in the Democratic Republic of Congo: a nationwide survey. Lancet 2006 Jan 7;367(9504):44-51.

(33) Coghlan B, Ngoy P, Mulumba F, Hardy C, Bemo VN, Stewart T, et al. Update on mortality in the Democratic Republic of Congo: results from a third nationwide survey. Disaster Med Public Health Prep 2009 Jun;3(2):88-96.

(34) UNICEF. Baby-friendly Hospital InitiativeTraining Materials (Updated 2009). Available at: <http://www.unicef.org/nutrition/index_24850.html>. Accessed November 20, 2010.

(35) WHO. Infant and Young Child Feeding Counselling: An integrated Course. 2006; Available at: <http://whqlibdoc.who.int/publications/2006/9789241594752_eng.pdf>. Accessed November 20, 2010.

(36) WHO, UNICEF. Baby-friendly hospital initiative : revised, updated and expanded for integrated care. Section
2, Strengthening and sustaining the baby-friendly hospital initiative: a course for decision-makers. 2009; Available at: <http://www.unicef.org/nutrition/files/BFHI_section_2_2009_eng.pdf>. Accessed November 20, 2010.

(37) WHO. Indicators for assessing infant and young child feeding practices. 2008; Available at: <http://www.who.int/child_adolescent_health/documents/9789241596664/en/index.html>. Accessed November 20, 2010.

(38) Montori VM, Guyatt GH. Intention-to-treat principle. CMAJ 2001 Nov 13;165(10):1339-1341.

(39) Williams RL. A note on robust variance estimation for cluster-correlated data. Biometrics 2000 Jun;56(2):645-646.

(40) Hayes RJ, Bennett S. Simple sample size calculation for cluster-randomized trials. Int J Epidemiol 1999 Apr;28(2):319-326.
