# Supplementary material for: Postpartum depressive symptoms following implementation of the 10 steps to successful breastfeeding program in Kinshasa, Democratic Republic of Congo: A cohort study
Source: PLoS Med. 2021 Jan 11;18(1):e1003465. doi: 10.1371/journal.pmed.1003465 (PMC7799755; doi:10.1371/journal.pmed.1003465)
Supplement: S1 IPW Calculation — (DOCX) [file pmed.1003465.s002.docx]

**Supplementary File 2: Postpartum depressive symptoms following implementation of the ten steps to successful breastfeeding program in Kinshasa, Democratic Republic of Congo: a cohort study**

Robert A. Agler, Paul N. Zivich, Bienvenu Kawende, Frieda Behets, Marcel Yotebieng

*Inverse Probability of Treatment Weights*

Inverse probability weights (IPW) were used to estimate the average causal effect (or the total effect) of Steps 1-9 and Steps 1-10 on EPDS scores at week 14, depression at week 14, any breastfeeding difficulties at week 10, and number of breastfeeding difficulties at week 10 compared to the standard of care. The average causal effect is defined as the prevalence of postpartum depression under the scenario where all mothers received steps 1-9 (or 1-10) compared to the prevalence of postpartum depression where all mothers had received the standard of care. This can be written as $E\left[ Y^{a=a'} \right]-E[Y^{a=0}]$ where $E[Y^{a}]$ is the average value of the outcome for individuals had they *all* been observed under treatment $a$. To estimate this quantity, inverse probability of treatment weights (IPTW) construct a weighted pseudo-population where the observed confounders are no longer related to the intervention [1]. IPTW allow for estimation of the average causal effect of an intervention under the assumptions of conditional exchangeability, causal consistency, and positivity [2, 3]. Stabilized IPTW were calculated as

$$w_{i}^{a}=\frac{\Pr\left( A_{i}=a \right)}{\Pr\left( A_{i}=a| L_{i} \right)}$$

where $A$ is trial arm and $L$ is the set of confounders. Probabilities of intervention arms were generated from a multinomial logistic regression model. IPTW were additionally used to account for confounding of the intervention and EPDS outcomes in the mediation analyses.

*Inverse Probability Weights for Mediation*

To estimate the controlled direct effect, IPW were used to account for confounders of the mediation-outcome relationship [4, 5]. The controlled direct effect corresponds to the change in depression or EPDS scores if the breastfeeding difficulties had been uniformly set to the same level in the population and BFHI implementations were compared to the standard of care [4]. IPW for mediation by the number of difficulties at week 10 took the following form

$$w_{i}^{m}=\frac{f(M_{i}=m|A_{i})}{f\left( M_{i}=m | A_{i}, L_{i}, W_{i} \right)}$$

where $f\left( \cdot\right)$ is the probability mass function, $M$ is the number of breastfeeding difficulties, and $W$ is clinic experience measures. The probability mass function comes from a Poisson model, since number of breastfeeding difficulties is a count data. For any reported difficulties, IPW for mediation were calculated as

$$w_{i}^{m}=\frac{\Pr\left( M_{i}=m \right|A_{i})}{\Pr\left( M_{i}=m| A_{i}, L_{i}, W_{i} \right)}$$

with probabilities generated from a logistic regression model.

*Inverse Probability of Censoring Weights*

Inverse probability of censoring weights (IPCW) were used to relax the assumption that censoring / loss-to-follow-up was non-informative [6, 7]. IPCW instead assume that censoring is non-informative conditional on the variables included in the weight model. IPCW were constructed using a logistic regression model predicting censoring as a function of trial arm, breastfeeding difficulties, demographics, and clinic experience. Weights for weeks 10 and 14 were calculated separately. Weights were calculated as

$$w_{i}^{c}=\frac{\Pr\left( C_{i}^{10}=1|A_{i}, M_{i} \right)}{\Pr\left( C_{i}^{10}=1| A_{i}, M_{i}, L_{i} \right)}\times\frac{\Pr\left( C_{i}^{14}=1|A_{i}, M_{i} C_{i}^{10}=0 \right)}{\Pr\left( C_{i}^{14}=1| A_{i}, M_{i}, L_{i}, C_{i}^{10}=0 \right)}$$

To combine weights, IPW were multiplied together. Weights for mediation analyses took the following forms; mediation IPW: $w_{i}^{m}$, mediation IPW with IPTW : $w_{i}^{m} \times w_{i}^{a}$, and mediation IPW with IPTW and IPCW: $w_{i}^{m} \times w_{i}^{a}\times w_{i}^{c}$. For outcomes measured at week 10 (number of difficulties breastfeeding and any difficulties breastfeeding), only the IPCW for week 10 were used.

*Marginal Structural Models*

Weights are used to estimate the parameter(s) of specified marginal structural models. To estimate the average causal effect of the interventions on EPDS scores at week 14, depression at week 14, any breastfeeding difficulties at week 10, and number of breastfeeding difficulties at week 10, the following marginal structural model was specified

$$E\left[ Y^{a} \right]=\alpha_{0}+\alpha_{1}a'+ \alpha_{2}a''$$

where $\alpha_{1}$ is the average causal effect comparing steps 1-9 to the standard of care and $\alpha_{2}$ is the average causal effect comparing steps 1-10 to the standard of care.

To estimate the controlled direct effect with mediation by the number of breastfeeding difficulties are week 10, the following marginal structural model was specified

$$E\left[ Y^{am} \right]=\alpha_{0}+\alpha_{1}a'+ \alpha_{2}a''+ \alpha_{3}m+ \alpha_{4}m^{2}+ \alpha_{5}a'm+ \alpha_{6}a'm^{2}+ \alpha_{7}a''m+ \alpha_{8}a''m^{2}$$

where the controlled direct effect was defined as $\alpha_{1}+ \alpha_{5}m+ \alpha_{6}m^{2}$ for steps 1-9 and $\alpha_{2}+ \alpha_{7}m+ \alpha_{8}m^{2}$ for steps 1-10. The number of difficulties was modeled as a quadratic term since there was indication the relationship was not linear. To estimate the controlled direct effect with mediation by the any difficulties breastfeeding at week 10, the following marginal structural model was specified

$$E\left[ Y^{am} \right]=\alpha_{0}+\alpha_{1}a^{'}+ \alpha_{2}a^{''}+ \alpha_{3}m+ \alpha_{4}a^{'}m+\alpha_{5}a''m$$

where the controlled direct effect for Step 1-9 is $\alpha_{1}+ \alpha_{4}m$ and for Step 1-10 is $\alpha_{2}+ \alpha_{5}m$. Confidence intervals for marginal structural models were calculated using robust variance estimators. To account for the clustered nature of the data, the clinic mother-infants pairs attended was used as the cluster-level for the robust variance.

**References:**

1. Robins JM, Hernan MA, Brumback B. Marginal structural models and causal inference in epidemiology. Epidemiology. 2000;11(5):550-60. Epub 2000/08/24. PubMed PMID: 10955408.

2. Hernán MA, Robins JM. Estimating causal effects from epidemiological data. Journal of Epidemiology and Community Health. 2006;60(7):578-86. doi: 10.1136/jech.2004.029496.

3. Hernan MA, Robins JM. Causal Inference. Boca Raton: Chapman & Hall/CRC; Forthcoming.

4. Robins JM, Greenland S. Identifiability and exchangeability for direct and indirect effects. Epidemiology. 1992:143-55.

5. VanderWeele TJ. Marginal Structural Models for the Estimation of Direct and Indirect Effects. Epidemiology. 2009;20(1):18-26. doi: 10.1097/EDE.0b013e31818f69ce. PubMed PMID: 00001648-200901000-00006.

6. Howe CJ, Cole SR, Lau B, Napravnik S, Eron JJ, Jr. Selection Bias Due to Loss to Follow Up in Cohort Studies. Epidemiology (Cambridge, Mass). 2016;27(1):91-7. doi: 10.1097/EDE.0000000000000409. PubMed PMID: 26484424.

7. Robins JM, Finkelstein DM. Correcting for noncompliance and dependent censoring in an AIDS Clinical Trial with inverse probability of censoring weighted (IPCW) log-rank tests. Biometrics. 2000;56(3):779-88. Epub 2000/09/14. PubMed PMID: 10985216.
