# Supplementary material for: Postpartum depressive symptoms following implementation of the 10 steps to successful breastfeeding program in Kinshasa, Democratic Republic of Congo: A cohort study
Source: PLoS Med. 2021 Jan 11;18(1):e1003465. doi: 10.1371/journal.pmed.1003465 (PMC7799755; doi:10.1371/journal.pmed.1003465)
Supplement: S1 Sensitivity analyses — (DOCX) [file pmed.1003465.s004.docx]

**Supplementary File 4: Postpartum depressive symptoms following implementation of the ten steps to successful breastfeeding program in Kinshasa, Democratic Republic of Congo: a cohort study.**

Robert A. Agler, Paul N. Zivich, Bienvenu Kawende, Frieda Behets, Marcel Yotebieng

To assess whether different results were obtained by how breastfeeding at 10-weeks was measured, we adjusted for the proportion of feedings that were breast feeding within the past week at week 10 postpartum. This definition is different from the main analyses, that included exclusive breastfeeding at week 10 in the inverse probability of mediation weight model. Results are presented below.

| **Supplemental Table 3.1: Estimated controlled direct effects mediated through any difficulty breastfeeding at week 10** | | | | | | | | | |
| --- | --- | --- | --- | --- | --- | --- | --- | --- | --- |
|  |  |  | Steps 1-9 | | |  | Steps 1-10 | | |
|  |  |  | Point estimate | 95% CL | p |  | Point estimate | 95% CL | p |
| EPDS score* | | |  |  |  |  |  |  |  |
|  | Mediation IPW ‡ | |  |  |  |  |  |  |  |
|  |  | No difficulty | -1.73 | (-4.32, 0.85) | 0.189 |  | 1.67 | (-0.76, 4.11) | 0.178 |
|  |  | At least one difficulty | -3.51 | (-4.52, -2.48) | <0.001 |  | -5.11 | (-6.09, -4.12) | <0.001 |
|  | With IPTW# | |  |  |  |  |  |  |  |
|  |  | No difficulty | -1.04 | (-5.09, 3.01) | 0.614 |  | 2.14 | (-0.21, 4.49) | 0.074 |
|  |  | At least one difficulty | -3.32 | (-4.44, -2.20) | <0.001 |  | -5.19 | (-6.27, -4.10) | <0.001 |
|  | With IPTW & IPCW | |  |  |  |  |  |  |  |
|  |  | No difficulty | -1.73 | (-4.98, 1.51) | 0.295 |  | 1.97 | (-0.48, 4.42) | 0.116 |
|  |  | At least one difficulty | -3.53 | (-4.68, -2.37) | <0.001 |  | -5.35 | (-6.46, -4.24) | <0.001 |
| Probable depression† | | |  |  |  |  |  |  |  |
|  | Mediation IPW ‡ | |  |  |  |  |  |  |  |
|  |  | No difficulty | -0.03 | (-0.13, 0.08) | 0.653 |  | -0.01 | (-0.14, 0.02) | 0.899 |
|  |  | At least one difficulty | -0.14 | (-0.22, -0.06) | 0.001 |  | -0.16 | (-0.23, -0.08) | <0.001 |
|  | With IPTW# | |  |  |  |  |  |  |  |
|  |  | No difficulty | 0.01 | (-0.17, 0.19) | 0.933 |  | -0.05 | (-0.15, 0.06) | 0.389 |
|  |  | At least one difficulty | -0.13 | (-0.23, -0.04) | 0.006 |  | -0.15 | (-0.24, -0.06) | 0.002 |
|  | With IPTW & IPCW | |  |  |  |  |  |  |  |
|  |  | No difficulty | -0.02 | (-0.17, 0.13) | 0.790 |  | -0.05 | (-0.15, -0.06) | 0.359 |
|  |  | At least one difficulty | -0.15 | (-0.24, -0.06) | 0.002 |  | -0.16 | (-0.26, -0.07) | <0.001 |
| 95% CI: 95% confidence interval. IPW: inverse probability weight, IPTW: inverse probability of treatment weight, IPCW: inverse probability of censoring weight. Any breastfeeding difficulties was at least one self-reported difficulty of breastfeeding at week 10 | | | | | | | | | |
| * The marginal structural model for EPDS was modeled as linear-Poisson, where point estimates correspond to differences in counts. | | | | | | | | | |
| † The marginal structural model for probable depression was linear-binomial. Point estimates for these results correspond to prevalence differences. Probable depression was defined as an EPDS score of at least 13 at 14 weeks post-partum. | | | | | | | | | |
| ‡ Mediation IPW used only the IPW weights calculated for any difficulties breastfeeding. Mediation IPW were conditional on age, education, marital status, previous children, prior miscarriage / abortion / stillbirth, not wanting additional children, prior experiences of domestic violence, home ownership, flush toilet facility, proportions of feedings that were breastfeeding, and whether they felt humiliated / intimidated / humiliated by delivery room nurses | | | | | | | | | |
| # With IPTW is mediation IPW multiplied by IPTW. IPTW for the intervention group were conditional on age, education, marital status, previous children, prior miscarriage / abortion / stillbirth, not wanting additional children, prior experiences of domestic violence, home ownership, and flush toilet facility. | | | | | | | | | |
| ** With IPTW & IPCW includes mediation IPW, IPTW, and IPCW. IPCW were conditional on age, education, marital status, previous children, prior miscarriage / abortion / stillbirth, not wanting additional children, prior experiences of domestic violence, home ownership, flush toilet facility, and given their baby right after delivery. | | | | | | | | | |

| **Supplemental Figure 3.1: Estimated controlled direct effects of EPDS score at 14-weeks post-partum mediated through number of difficulties breastfeeding at week 10** |
| --- |
| 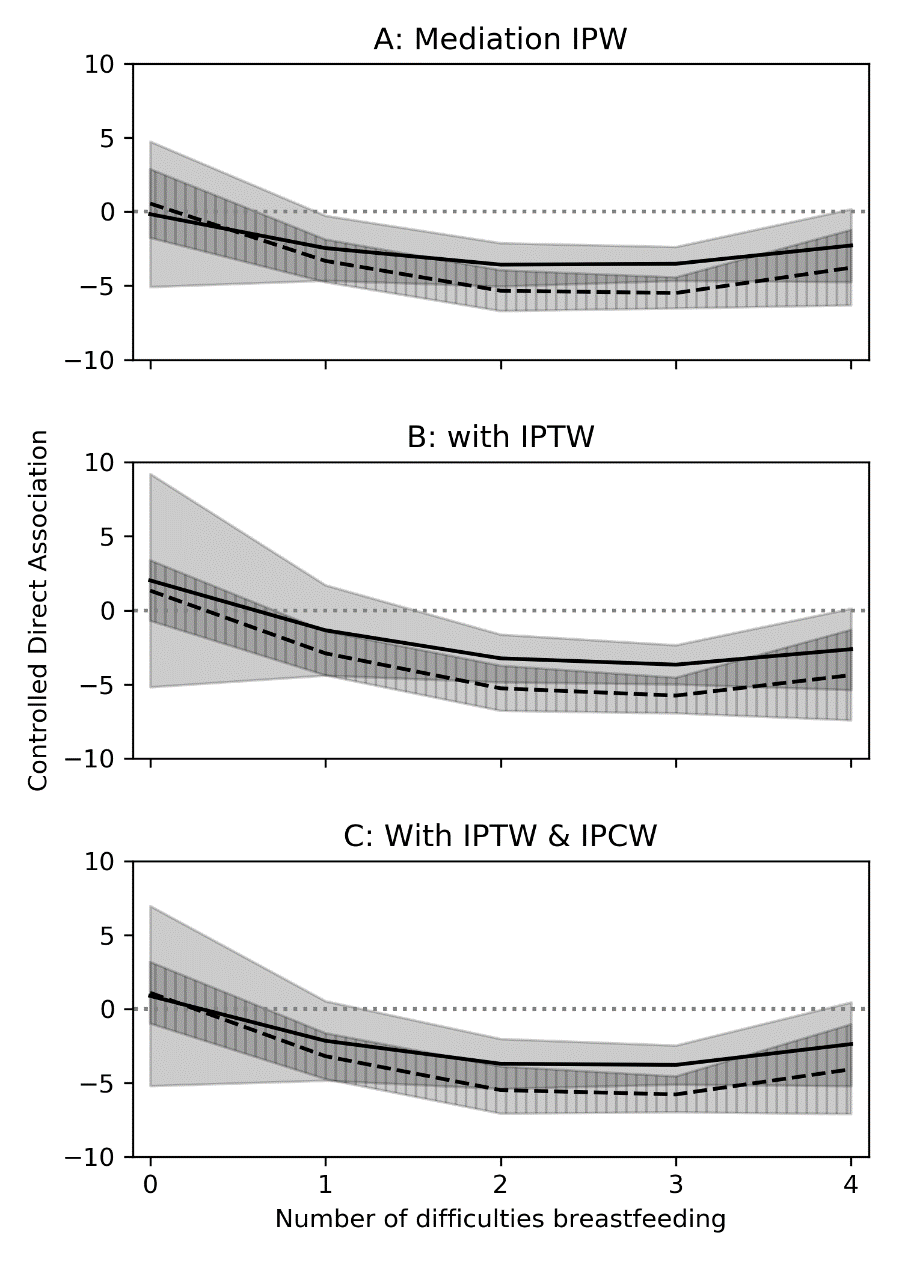 |
| 95% CI: 95% confidence interval. IPTW: inverse probability of treatment weight, IPCW: inverse probability of censoring weight. Solid line and solid shaded indicated Steps 1-9 point estimate and confidence intervals, respectively. Dashed line and hatched region indicated Steps 1-10 point estimate and confidence intervals, respectively.  The marginal structural model for EPDS and number of difficulties modeled was modeled as linear-Poisson, where point estimates correspond to differences in counts. |

| **Supplemental Figure 3.2: Estimated controlled direct effects of probable depression at 14-weeks post-partum mediated through number of difficulties breastfeeding at week 10** |
| --- |
| 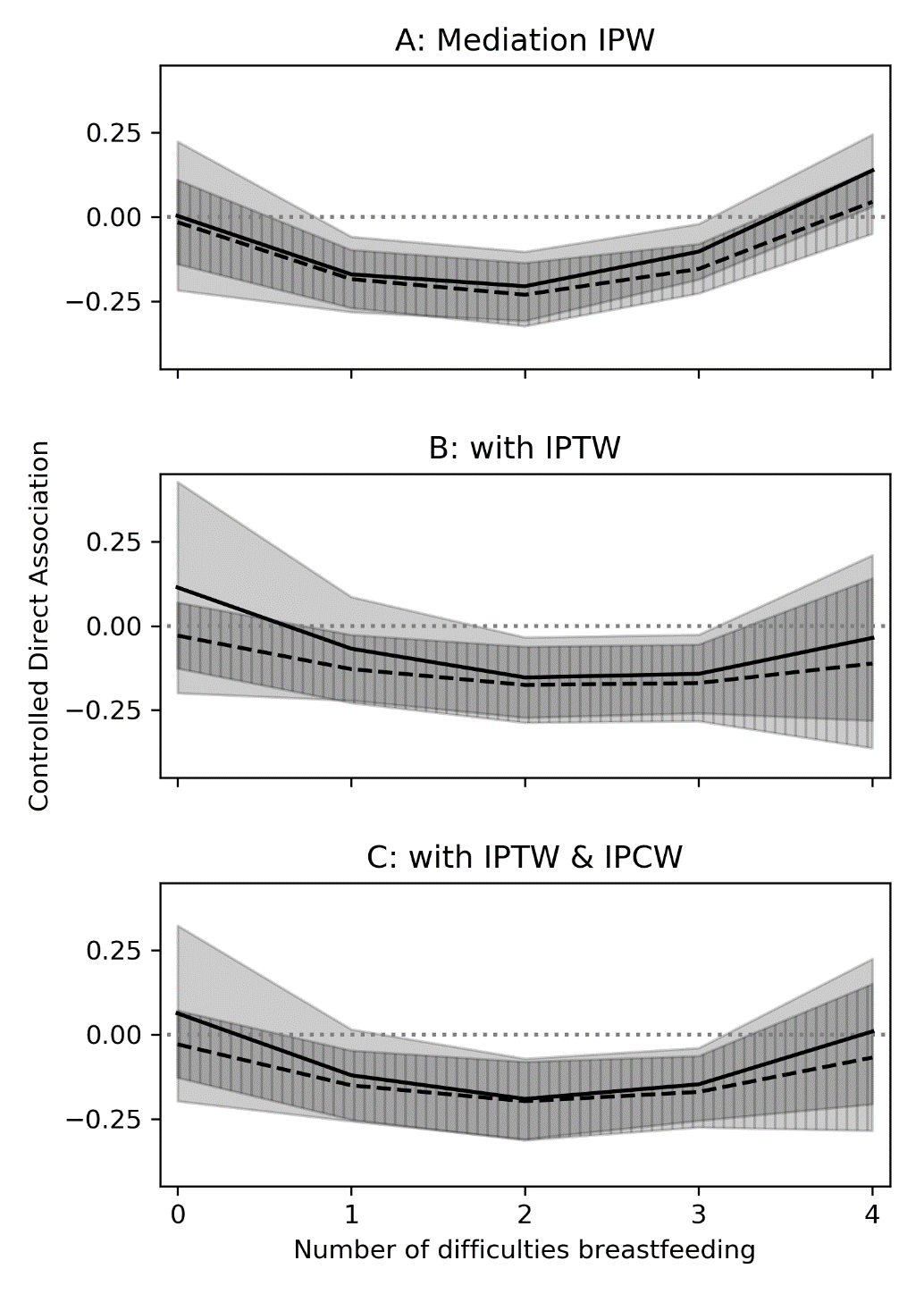 |
| 95% CI: 95% confidence interval. IPTW: inverse probability of treatment weight, IPCW: inverse probability of censoring weight. Solid line and solid shaded indicated Steps 1-9 point estimate and confidence intervals, respectively. Dashed line and hatched region indicated Steps 1-10 point estimate and confidence intervals, respectively.  The model for number of difficulties modeled was modeled as linear-Poisson. The marginal structural model for probable depression was linear binomial, with point estimates corresponding to prevalence differences. Probable depression was defined as an EPDS score of at least 13 at 14 weeks post-partum. |
