# Supplementary material for: Postpartum depressive symptoms following implementation of the 10 steps to successful breastfeeding program in Kinshasa, Democratic Republic of Congo: A cohort study
Source: PLoS Med. 2021 Jan 11;18(1):e1003465. doi: 10.1371/journal.pmed.1003465 (PMC7799755; doi:10.1371/journal.pmed.1003465)
Supplement: Attachment — Submitted filename: agler.pdf [file pmed.1003465.s007.pdf]

This paper describes a cluster trial of the effect of a breast feeding promotion on depressive symptoms.

I was asked for a statistical report and I interpret that to include all aspects of the design and conduct of the study.

# Points of detail

**Page 1** I think in the abstract it would be better to use the phrase depressive symptoms (as in the title) or if the categorical outcome is meant then probably depression since this is not a diagnostic interview.

**Page 5** Why choose a quadratic model for age and breast feeding difficulties? This may well be justified but we do not know why or whether this was theory– or data–driven.

**Page 5** The issue of missing outcomes appears to be discussed here but not of missing covariates. The supplementary file does suggest that some covariates had missingness.

# Points of more substance

## Reporting detail

Presenting secondary analysis of trials is an important way of making use of the time spent by the staff and participants but it does raise the question of how much detail is needed in the secondary report and I feel in this case we have too little detail. I do not think that the suggestions of the CONSORT guidelines are all fulfilled. It would be helpful to have a flow diagram of participants through the multiple time points of observation for instance.

## Cluster nature of trial

I may have missed this somewhere but I do not see any explicit mention of how the cluster structure was taken into account in the analysis. It would also help to clarify the number of clusters as it is not clear whether there are six per arm or six overall. I assume six per arm as two per arm would have been rather limiting.

### Use of baseline

Table 2 makes it clear that at baseline (what the authors refer to as enrollment) the arms were very different on the depressive symptom score with baseline medians of 8, 7 and 3. I do not see any mention of how this was taken into account. On the face of it such differences at enrollment will make interpretation challenging.

### Intention to treat and invalid data

On page 10 we learn that the results from one staff member were regarded as suspect. This sort of thing often happens in real life but I wonder whether it is better to treat these as missing rather than to impute the inverse of what was reported. I think we can assume the values were missing completely at random.

## Summary

Some issues which need some more work.

Michael Dewey
