# Supplementary material for: Postpartum depressive symptoms following implementation of the 10 steps to successful breastfeeding program in Kinshasa, Democratic Republic of Congo: A cohort study
Source: PLoS Med. 2021 Jan 11;18(1):e1003465. doi: 10.1371/journal.pmed.1003465 (PMC7799755; doi:10.1371/journal.pmed.1003465)
Supplement: Attachment — Submitted filename: Response to reviewers and editors comments_pnz.docx [file pmed.1003465.s008.docx]

July 13, 2020

Caitlin Moyer, Ph.D.

Associate Editor

PLOS Medicine

plosmedicine.org

Re: PMEDICINE-D-20-00294R1

Dear Editors,

Thank you for the opportunity to resubmit our manuscript, ‘*Implementing the "Implementation of Ten Steps to Successful Breastfeeding Program Reduces Postpartum Depressive Symptoms: additional outcomes from a cluster-randomized controlled trial in Kinshasa, Democratic Republic of Congo*”

Below, we provide a point-by-point response to the editors’ and reviewers’ comments.

**Requests from the editors:**

1. Title: Please revise the title to reflect that these are not the primary trial outcomes. Please also include information on the study population, we suggest: “Implementation of Ten Steps to Successful Breastfeeding Program and Postpartum Depressive Symptoms: additional outcomes from a cluster-randomized controlled trial in Kinshasa, Democratic Republic of Congo”

**Response:** We have updated the title as requested.

Line 1-4: “Implementation of Ten Steps to Successful Breastfeeding Program Reduces Postpartum Depressive Symptoms: additional outcomes from a cluster-randomized controlled trial in Kinshasa, Democratic Republic of Congo”

2. Throughout the manuscript: Because this manuscript reports on non-registered outcomes from the trial, please remove the use of causal language such as “effect of” “was mediated by”

**Response:** We strongly disagree that solely on the basis of that the outcome was not a registered trial outcome a causal interpretation of the result is not possible. The parameter of interest in our study is the average causal effect (total effect) of BFHI on depressive symptoms as measured through EPDS, and the controlled direct effect. These parameters were designated prior to the data analysis, and the variables included in the analytic approaches was determined by these aims. Replacing “cause” and “mediate” throughout the manuscript obfuscates the original goal of the analysis. Instead the aim of our study become ambiguous and liable to misinterpretation. Without the explicit language, cause and words like ‘association’ are needlessly conflated because non-registered outcomes were used. The conflation of cause and association leads to misinterpretation of findings more widely in the literature. We understand the editors concern to reduce statistically significance fishing in randomized trials for non-registered outcomes, but this is not how our analyses were approached.

While there is no guarantee the average causal effects can be identified, this similarly applies to even randomized trials for registered outcomes due to chance imbalances between randomized groups. In our work, EPDS was administered independently of randomization to all participants seen at 14 weeks. Our thorough analyses show consistent results even after accounting for informative censoring (lost-to-follow-up) and potential imbalances (via inverse probability of treatment weights). There are threats to the causal interpretations and we detail the limitations that threaten this interpretation. Despite the limitations present, the original aim of the study remained.

Our arguments for referring to our estimates as causal and mediation are further explicated in Hernan’s “The C-Word: Scientific Euphemisms Do Not Improve Causal Inference From Observational Data” *AJPH* 2020. We note that Hernan’s argument is primarily in the context of observational studies, but the argument similarly applies to randomized trials for non-registered outcomes.

3.Prospective analysis plan: Your outcomes differ from the registered trial protocol (NCT01428232). Did your study have a prospective protocol or analysis plan? Please state this (either way) early in the Methods section.

a) If a prospective analysis plan (from your funding proposal, IRB or other ethics committee submission, study protocol, or other planning document written before analyzing the data) was used in designing the study, please include the relevant prospectively written document with your revised manuscript as a Supporting Information file to be published alongside your study, and cite it in the Methods section. A legend for this file should be included at the end of your manuscript.

b) If no such document exists, please make sure that the Methods section transparently describes when analyses were planned, and when/why any data-driven changes to analyses took place.

**Response**: *We have also clearly stated that PPD was not a registered trial outcomes (Line 133).*

4. Data availability statement: Thank you for your willingness to share your data. Please provide the supplemental information spreadsheet (S4 data) as a separate file (not Zipped with SAS file) and include a descriptive legend explaining the column headers (variables).

**Response:** We have provided the data (as a .csv) separately from the SAS file. The data dictionary has also been provided as the descriptive legend (we note that we also provide the legend at the top of the SAS file)*.*

5. Abstract: Please structure your abstract using the PLOS Medicine headings (Background, Methods and Findings, Conclusions).

**Response:** We have re-structured the abstract headings.

6. Abstract: Please describe the population demographics, including number of participants and the years during which the study took place.

**Response:** We have added this information to the abstract.

Lines 24-27: “We used data from a breastfeeding promotion trial of all women who gave birth to a healthy singleton between May 24 and August 25, 2012 in one of the six facilities comparing different BFHI implementations (Steps 1-9, Steps 1-10) to the standard of care randomized by facility in Kinshasa, Democratic Republic of Congo.”

Lines 32: “903 mother-infant pairs were included in the analysis.”

7. Abstract: Line 26 (and throughout the manuscript): Please remove all references to causality, as these appear to be the post-hoc (additional) outcomes rather than the planned registered trial outcomes.

***Response:*** *Please refer to our previous argument for why we would prefer to refer to the parameters as causal effects and controlled direct effects. To prevent ambiguity, we request that the editors allow for the use of ‘causal’ in the manuscript. If not, please provide guidance on how to clearly explain the parameters of interest.*

8.Abstract: Line 29-31 (and throughout manuscript): Please quantify the main results with both 95% CIs and p values.

**Response:** *We have presented both 95% CI and p-values where appropriate.*

9.Abstract: In the last sentence of the Methods and Findings section, please describe the main limitation(s) of the study's methodology.

**Response:** *We have added a sentence at the end of the Findings.*

*Lines 37: “However, the results are based on two hospitals randomized to each group.”*

10.Abstract Conclusions: Please address the study implications without overreaching what can be concluded from the data; the phrase "In this study, we observed ..." may be useful. Your results do not address infant health, so please revise or remove the following sentence: “The BFHI steps may improve infant health due to increased exclusive breastfeeding and maternal health due to reduced stress associated with infant feeding.”

**Response:** *We have removed the sentence and reframed the conclusion.*

*Lines 38-43: “In this study, we observed that depressive symptoms were reduced in the groups implementing Steps 1-9 or 1-10 relative to the standard of care, with implementation of Steps 1-10 resulting in the largest decrease. This reduction was mediated through attenuation of breastfeeding difficulties. PPD has a huge negative impact on the mother, her, partner, and the baby, with long lasting consequences. This newly found additional benefit of BFHI steps call for renewed effort to scale its implementation globally.”*

11.Author Summary: At this stage, we ask that you include a short, non-technical Author Summary of your research to make findings accessible to a wide audience that includes both scientists and non-scientists. The Author Summary should immediately follow the Abstract in your revised manuscript. This text is subject to editorial change and should be distinct from the scientific abstract. Please see our author guidelines for more information:

***Response:*** *We have included an author summary after the abstract as requested on lines 44-64.*

12. Methods: Line 104-105: Trial registry: This manuscript does not appear to describe the primary or secondary outcomes of the trial as registered. Please clarify and explain the discrepancy. If the outcomes were not prespecified in the protocol, please indicate that they were post hoc and explain why they were added. Post hoc comparisons should be presented as hypothesis generating rather than conclusive. Please include the study protocol document and analysis plan, with any amendments, as Supporting Information to be published with the manuscript if accepted.

***Response:*** *This is correct; depression was not included in registered trial outcomes and our analysis is post-hoc. We have made this clear in the manuscript.*

*Line 133-134: “Depression was not included in registered trial outcomes.”*

13. Methods: Lines 111-118: Please specify whether informed consent was written or oral.

***Response:*** *Inform consent was written. We have specified this in the Methods.*

*Line 177: “All participants provided written informed consent.”*

14.Methods: Line 125-126: Although published elsewhere, please briefly describe the SOC condition.

***Response:*** *We have added a description of the SOC.*

*Lines 160-162:* *“In facilities randomized to standard of care, besides study briefing prior to randomization, nothing else was provided aside from routine care.”*

15.Methods: Please provide the EPDS as a supporting information file, including information on scoring.

***Response:*** *We have added this as an additional supplementary file.*

16.Results: Line 207-208: Here, and throughout, please remove causal language and temper statements such as “Among both intervention groups, the effects of the 207 BFHI on depression were mediated

208 through breastfeeding difficulties.”

***Response:*** *Please see our previous response to these comments on removing causal language. If we can’t refer to this as “average total effect” and “controlled effect” or “mediated effect”, we welcome the editors’ suggestion for how to describe and interpret these estimates that still clearly delineates variables on the causal path. We find “association” to be vague and not indicate that difficulties breastfeeding is on the causal path between BFHI and EPDS scores.*

17. Discussion: Please present and organize the Discussion as follows: a short, clear summary of the article's findings; what the study adds to existing research and where and why the results may differ from previous research; strengths and limitations of the study; implications and next steps for research, clinical practice, and/or public policy; one-paragraph conclusion.

***Response:*** *We have reframed the discussion as suggested, thanks.*

18. Checklist: Please complete the CONSORT checklist, and ensure that all components of CONSORT are present in the manuscript. Please ensure that the study is reported according to the CONSORT guideline, and include the completed CONSORT checklist as Supporting Information. When completing the checklist, please use section and paragraph numbers, rather than page numbers.

Please add the following statement, or similar, to the Methods: "This study is reported as per the Consolidated Standards of Reporting Trials (CONSORT) guideline (S1 Checklist)."

***Response:*** *We have added the CONSORT checklist to the supplementary materials*

*Line 235-236: “This study is reported as per the Consolidated Standards of Reporting Trials (CONSORT) guideline (S1 File).”*

19. Table 2: The groups appear to differ, for example in the measure “baby given right after birth”- please comment on whether this difference is a limitation or could contribute to results.

***Response:*** *One of the aim of the parent study was initiation of breastfeeding within one hour of birth. this require that the baby be given to the mother immediately following birth. So the difference was expected. Therefore, this difference is a results (effect) of the intervention itself and is not necessary for adjustment. As such, differences by this factor are not (and should not be) controlled in the total effect analyses. However, it does present as a potential confounder for the controlled direct effect. For this reason, “baby given right after birth” was included in the inverse probability weights for the mediation model. This method has been well detailed as an approach to account for imbalances by observed variables. Nonetheless, we discussed the limitation that groups may not be balanced for all baseline characteristics (Line 372-379). However, the statistical methods used are meant to account for the imbalances in variables included in the inverse probability weighting calculations.*

20. Table 4 and Table 5 (and relevant section of the Results section text): Please provide both the 95% CIs and p values for all analyses.

***Response:*** *We have added columns of p-values and in the text as requested.*

Comments from the reviewers:

**Reviewer #1:**

Page 1 I think in the abstract it would be better to use the phrase depressive symptoms (as in the title) or if the categorical outcome is meant then probably depression since this is not a diagnostic interview.

***Response****: We have updated the language to refer to depressive symptoms as suggested.*

Page 5 Why choose a quadratic model for age and breast feeding difficulties? This may well be justified but we do not know why or whether this was theory– or data–driven.

***Response****: The choice of quadratic models for age and breastfeeding difficulties was based on the data. To allow for greater flexibility, we have updated the propensity score models to model age with restricted quadratic splines instead (lines 205-206).*

*For breastfeeding difficulties, quadratic terms were selected since they improved the fit of the marginal structural model. However, the addition of cubic terms resulted in model convergence issues, most notably in the linear-binomial. Therefore, we were limited to examining quadratic models for number of breastfeeding difficulties.*

Page 5 The issue of missing outcomes appears to be discussed here but not of missing covariates. The supplementary file does suggest that some covariates had missingness.

***Response****: We believe the major threat of missing data is potentially informative censoring (i.e. missing data on number of breastfeeding difficulties at 10-weeks and EPDS at 14-weeks). This is why we chose to use inverse probability of censoring weighting. As the reviewer notes, there is other sources of missing data as indicated in Table 1. The extent of missing data for these variables is small (<5%) and therefore will have small impacts. The potential benefits of multiple imputation or inverse probability of missing weighting for these variables will be negligible or minor due to the small amount of missing data. As a result, we chose to focus on censoring only instead.*

Presenting secondary analysis of trials is an important way of making use of the time spent by the staff and participants but it does raise the question of how much detail is needed in the secondary report and I feel in this case we have too little detail. I do not think that the suggestions of the CONSORT guidelines are all fulfilled. It would be helpful to have a flow diagram of participants through the multiple time points of observation for instance.

***Response****: We have updated our manuscript to correspond to the CONSORT guidelines. Furthermore, we have added a new figure (Figure 1) which provides a flow diagram for the participants.*

I may have missed this somewhere but I do not see any explicit mention of how the cluster structure was taken into account in the analysis. It would also help to clarify the number of clusters as it is not clear whether there are six per arm or six overall. I assume six per arm as two per arm would have

been rather limiting.

***Response****: There was only two per arm, which we agree is rather limiting. We had attempted to account for the clustering structure through GEE. However, the clinic-level estimated variance was substantially narrower than the individual-level estimated variance in some scenarios (it was also wider in others). GEE is known to have poor performance and can be biased when the number of clusters is below 40 (Mancl & DeRouen Biometrics 2001). In fact, for a small amount of clusters, the variance can be downwardly biased. For these theoretical and practical reasons, we believe that the GEE results are more misleading than ignoring the clustering of the data.*

*While mixed-effect models are another option, our parameters of interest are the marginal effects and marginal controlled direct effects. Mixed-effect models are no longer advised for the marginal estimates (Hubbard et al. Epidemiology 2010). Therefore, those approaches were excluded.*

*We have clarified this and the limitation of our approach further.*

*Lines 354-356: “Despite being a randomized trial, the small number of clusters precluded accounting for the clustering in the variance. Therefore, results may be optimistic in terms of precision (as indicated by the confidence limit differences).”*

Table 2 makes it clear that at baseline (what the authors refer to as enrollment) the arms were very different on the depressive symptom score with baseline medians of 8, 7 and 3. I do not see any mention of how this was taken into account. On the face of it such differences at enrollment will make

interpretation challenging.

***Response****: The depressive symptom scores in Table 1 are not the baseline scores, but rather the scores at 14-weeks. We have updated the row names to indicate that the scores were at 14-weeks. Unfortunately, baseline depressive symptoms were not collected, as noted in the limitations (lines 390-391).*

On page 10 we learn that the results from one staff member were regarded as suspect. This sort of thing often happens in real life but I wonder whether it is better to treat these as missing rather than to impute the inverse of what was reported. I think we can assume the values were missing completely at

random.

***Response****: Originally, we had included participants assessed by that reviewer in the main results and conducted a sensitivity analysis excluding those participants. In order to prevent readers’ confusion and the assumption of those values being missing completely at random being reasonable, we have changed the main results to exclude participants assessed by that reviewer. We no longer include results with those participants in the sensitivity analyses.*

**Reviewer #2:**

In this article, the authors describe a study examining the impact of the Baby-Friendly Hospital Initiative (BFHI) on postpartum depression at 14 weeks postpartum for a sample of mothers in Kinshasa, Democratic Republic of the Congo.

This study represents a novel area of study. While previous studies have found the BFHI improves breastfeeding initiation and duration, and effective breastfeeding lowers mothers' risk of depression, this is the first to show that breastfeeding support--especially when there are problems--lowers mothers' risk of depression.

As enthusiastic as I am about this topic, there are several areas where some clarification is needed.

Introduction

The authors work with the premise that both breastfeeding difficulties and mental illness subject mothers to shame and stigmatization. True, but these are not the only factors at play here. And many would argue (include some very vocal social media groups) that BFHI, and breastfeeding support in general, causes shame and stimatization. How does the BFHI lessen the impact of these? I note that this is a secondary analysis. However, I'd to see the authors show a somewhat deeper understanding of the complex relationship between depression and breastfeeding. Simply hypothesizing about possible shame and stigmatization only scratches the surface of possible underlying mechanisms and these seems to be the main mechanisms offered as an explanation for these findings.

Rather than something like shame as the major factor, there is likely a physiological mechanism underlying these results. For example, social support increases oxytocin, which lowers risk of depression (by downregulating the stress system). This helps with both breastfeeding and depression. In contrast, breastfeeding pain suppresses oxytocin, increasing both breastfeeding problems and depression risk. Skin-to-skin contact increases oxytocin and directly reduces stress. None of the steps does anything regarding shame and stigmatization. Nor do they directly address "common postpartum stressors" (discussion section), especially when implemented in the hospital. Many of those "common stressors" have nothing to do with breastfeeding and are therefore not addressed by BFHI.

For the purposes of this manuscript, I'd like to see the authors reference the wider PPD literature, particularly with regard to breastfeeding. I'd also suggest examining research in psychoneuroimmunology. I'd suggest the work of Janice Kiecolt-Glaser, Lisa Christian, Kerstin Uvnas-Moberg, and Kathleen Kendall-Tackett.

***Response.*** *We thank the reviewer for the suggestion. We have added the following to the introduction:*

*Line 108-111: “Further,* *by increasing early initiation (within one hour of birth) and prolonged exclusive breastfeeding,[13] BFHI interventions likely are associated with increased levels of oxytocin among mothers. Higher levels of oxytocin are associated with lower reported breastfeeding difficulties and improved maternal mood [14, 15].”*

Please note a significant typo on Table 2. The abbreviation for the Edinburgh Postnatal Depression Scale is EPDS (not EPSD).

***Response:*** *We thank the reviewer for catching this error and this has been corrected.*

Interesting that exclusive breastfeeding rates seemed to be lower in mothers who received the full BFHI rather than steps 1-9. Can the authors comment on that?

Also, how do the authors explain that the BFHI seemed to be related to MORE problems than SOC. That would be a somewhat disturbing result. Also curiously, the EPDS scores appeared to be higher in the BFHI groups (Steps 1-9) when there were no breastfeeding problems.

***Response****: The reviewer is correct to point this out as a paradoxical observation. In the paper reporting registered primary outcomes of the trial, we speculated that “The lower prevalence of exclusive breastfeeding in the steps 1–10 group suggest that the materials used might not have provided the accurate or necessary messages for this setting, or that engagement of family members might have led to misunderstandings or incorrect advice that were not sufficiently countered by group counselling from nurses during well-child visits.”[1]*

*As for the observed increase in EPDS scores when all individuals in the population are ‘set’ to have no difficulties breastfeeding, the increase is not robust in the updated analyses. It is quite imprecise and lessens when exclusive breastfeeding at 10 weeks is modeled differently (see supplement 3). We provide an updated description on lines 261-264 and 369-374.*

Something else I'd like the authors to address is that what the mothers were identifying as problems all seem to be related to having "too much" milk. The problems that plague mothers in industrialized countries (sore nipples, not enough milk) are rare. This suggests that the results will not easily generalize to industrialized countries.

***Response:*** *Breastfeeding is the norm in Kinshasa. 9 in 10 women will breastfeed their baby through their first year. Given the majority of participant (at least in intervention groups) were exclusively breastfeeding, it’s not surprising that they are producing enough milk, particularly given that they are not pumping the excess production for storage. But from that to conclude result is or is not generalizable to high income countries depend on the hypothesized mechanism behind the observed effect. If. as our findings indicate, the effect is mediated through attenuation of breastfeeding difficulties, one will expect that to be true no matter the context. Additionally, the total effect appears to partially be through the identification of those difficulties. Though, we agree that future work should assess this in different contexts.*

I've been around the BFHI for more than 20 years. It is not designed to help mothers with stress management (per Discussion section). I wouldn't even say that social support for the mother is the goal (although that happens indirectly). As the name implies, it's BABY-friendly. It's designed to set mothers and babies up for successful breastfeeding, partly by allowing natural processes to take place.

***Response:*** *We do agree the BFHI intent is not to help mothers with stress management. But given the observed impact on depressive symptoms and the fact that this effect appears to be mediated through breastfeeding difficulties, it is not a stretch to suggest that an unintended benefit of BFHI is that it reduces stressors. This is a new finding that add to the existing one that the reviewer described.*

I found the second paragraph of the Discussion to be somewhat horrifying. OF COURSE, BFHI is not set up to deal with any postpartum mental illness, but especially severe illness. Heaven forbid! I've been training hospitals and baby-friendly providers for 25 years on how to recognize the symptoms of depression so they can be addressed, but not by breastfeeding supporters unless they are also mental health providers. But I've never thought that simply being in a program like this was enough. And we should not represent it as such.

***Response:*** *We do agree with the reviewer that access to proper mental health care should be advocated for all in need. We also agreed that as stated the paragraph could lead to misinterpretation. We have reframed the entire paragraph to avoid any suggestion that we are advocating BFHI as alternative mental health care.*

I also don't like paragraph 3. The risks of depression on mom and baby sound somewhat trite to me. How about including that PPD causes misery for the woman, her partner, and her baby. It impacts her interaction style with her infant, and that's what causes the problems downstream for babies (particularly because it can lead to insecure attachments). In the face of such serious problems, talking about a mother's return to her pre-pregnancy weight seems incredibly trivial. It's the type of comment that tends to rankle the mothers I've interviewed in my various studies. They often comment on "clueless" HCPs and it is one more reason why they stay silent. And how important is that issue in the DRC? Seems a bit like a first-world problem to me.

***Response****: We have reframed this part of the discussion as suggested by the reviewer as well.*

I note a number of really old references in the Reference section. Quite a few from the 1990s and some not specific to PPD. Some are not even related to depression. I'd urge the authors to update their understanding of PPD particularly as it relates to breastfeeding.

***Response:*** *We thank the reviewer for these suggestions, in reframing the discussion we have updated the citations with more recent literature.*

One final point. The EPDS, the primary measure of depression in this study, is actually a screening tool, not something that should be used to diagnose. I know that a lot of people do use it for a lot things it was never intended to be, but the authors need to acknowledge this as a limitation. In the current manuscript, that is not even mentioned. They also did not indicate how they scored it. What was the cutoff score they used to define depressed vs. non-depressed? Or did they use the EPDS as a continuous variable? Why did they choose that cutoff or decide to score it as a continuous variable? The authors need to provide more details here as well.

***Response:*** *We thank the reviewer for this observation and have added this limitation to the discussion.*

*Lines 196-197: “The EPDS is a short 10-item screening tool to assess postpartum depression symptoms that has been…”*

*The cutpoint used is indicated at lines 199-200 as well as* the footnotes of both tables and figures that included the dichotomization of EPDS.

**Reviewer #3**

This manuscript is an important addition to the evaluation of BFHI implementation and the impact on maternal health. Postpartum depression is a complex issues and support is an important part of the treatment strategy. The strengths of this paper include the quasi-experimental design. It is appropriate to randomize the intervention by clinic rather than by individual. The measure of postpartum depression has been validated. The statistical analysis is well described. There are a few major issues that have not been covered by this manuscript:

BFHI treatment fidelity at intervention sites. Were all of the steps implemented consistently during the evaluation period? The study assumes that the Steps were implemented and the only measure in this study of study participant exposure to the steps what the one question about being given the baby right after birth or Step 4 but studies have shown that Step 6 "Giving newborn infants no food or drink other than breastmilk, unless medically indicated, and not accepting free or low-cost breastmilk substitutes, feeding bottles, or teats" being very important to exclusive breastfeeding at discharge.

***Response:*** *We understand the concern of the reviewer. As described in the previous reports from this study, free or low-cost breastmilk substitute and other feeding bottles, or teats are not allowed in DRC as a matter of national policy. In addition, the goal of the parent study was to look at a simplified way to implement BFHI (without certification), that can be easily scaled-up and our results show that this works well for the primary aim of BFHI (increase exclusive breastfeeding). We have added information the evaluation of the implementation of BFHI steps, lines 158-160.*

Comparability of Sites: It would be helpful to have a description of the six sites and by important descriptive (operational descriptors geographic setting, size of site, size of maternity staff, number of deliveries per year).

***Response:*** *within the words limits of a manuscript, it’s difficult to provide all the details. On Line 162, we have clearly pointed the reader to were to find information on the contextual breastfeeding practices and description of the setting.*

Inclusion and exclusion criteria are not discussed in the paper. Were preterm babies included? Were mothers and babies with medical conditions included?

***Response:*** *Line 171-174: we have included eligibility and exclusion criteria.*

Maternal History of depression was not included. Mothers who have a history of depression before becoming pregnant or during pregnancy are much more likely to score high on the postpartum depression inventory. These mothers need to be analyzed separately or excluded from analysis.

***Response:*** *We agree with the reviewer that though, the exposure (BFHI groups) was randomly assigned and thus prior depression is not expected to be related to BFHI groups in expectation, it is however, a threat to the mediation analyses though (since difficulties BF isn’t randomly assigned). It also could be a common cause since individuals weren’t randomized (some clinics might be in an area with higher depression prevalence). We have noted this in the limitations on lines 391.*

Mother's experience of BFHI steps Page 4, Ln 137-138. As mentioned above, only one step is assessed and does not reflect the entire BFHI experience. Mothers can report on Steps 4-10. You may not have been able to measure all of these steps but it should be acknowledged as a limitation in your discussion.

***Response:*** *Lines 158-160: we have added information on assessment of the implementation of Steps 1-9*

Page 4 ln 117 "Women were enrolled within two days of delivery and follow-up at 6, 10, 14, 18, and 24-weeks postpartum." Indicates longitudinal data collected on your population. Although postpartum depression is only collected at week 14, is breastfeeding exclusivity and breastfeeding difficulties collected at multiple times? If so, timing of the difficulties (2 days, 6 weeks vs 10 or 14 weeks) would be important to investigate. Were earlier difficulties mediators of depression or were later difficulties or no difference. Most breastfeeding difficulties which impact exclusive breastfeeding happen early.

***Response:*** *The reviewer is correct about the longitudinal data collection. Yes, we did collect data on breastfeeding difficulties at each time point. We agree that the timing of the difficulties would be important to investigate. But, we will reserve this for another manuscript.*

Table 2: Characteristics of women at study enrollment by intervention group (n=975). Two results were not discussed in the results or discussion sections but seem to be very relevant to this study. The first was Any breastfeeding difficulties, Step 1-10 sites participants reported 91% compared with the SOC 67% and Steps 1-9 sites 75%. Sites 1-10 may have identified more breastfeeding difficulties as a result of attending support sessions after discharge. The other result not discussed was Exclusive breastfeeding up to week 10 SOC site participants reported 117 (38%), the Steps 1-9 - 237 (65%) and Steps 1-10 - 178 (58%). It is interesting that Steps 1-9 participants had higher rates of exclusive breastfeeding than the Steps 1-10 site participants. This finding in conjunction with the breastfeeding difficulties findings need to be explored more in discussion as they may have related to the results.

***Response:*** *The reviewer is right for the fact that the level of breastfeeding difficulties is related to the study result as illustrated by the result of the controlled direct effect. We have expanded this description on lines 326-329.*

*As for the higher rate of exclusive breastfeeding among steps 1-9 compared to steps 1-10, this was discussed in previous work. Please see the response to reviewer 2.*

In Table 3: Reported breastfeeding difficulties at ten weeks post-partum (n=851), the most frequently cited breastfeeding difficulty for all three groups is Baby nursed too often with SOC-157 (58%), Steps 1-9-164 (54%) and Steps 1-10-161 (59%). This difficulty has not be a prominent difficulty in the literature and seems to be high in all three groups. At 14 weeks if might reflect a growth spurt and misunderstanding of baby developmental cues and growth needs by the mother which should be provided by a support group in the Steps 1-10 group. However even though breastfeeding difficulties are enumerated and used as a mediator, they are not discussed in the results nor the discussion. This is a major omission in this paper. Breastfeeding difficulties may provide information for future interventions on support of mothers. Depression is a multifaceted and multisensory concern. 'Nursing too often' may be a sign of an overwhelmed mother or a signal for outside support for

other activities so that mother can provide the nutrition to the baby without the distraction or distress with not fulfilling other family activities.

***Response:*** *We do agree with the reviewer that this paper open a new line of research regarding breastfeeding difficulties and depression and that a more detailed analysis of those difficulties, as it relate to their timing and their nature is needed. But including all of this in a single manuscript will make the paper too dense and difficult to read. We will be doing these analyses in proper details for another paper.*

**Reviewer #4**

An important paper in terms of assessing if BFHI with or without the community support group contact reduces maternal depression.

There are however some things that the authors should consider stating upfront. Since paper and findings are based on a "post-hoc" analyses of a trial data set, it should be stated clearly.

**Response**: We have stated this in the methods Line 133-134. We also indicate this information in the title of the paper and abstract.

Title: Since this paper reports on objectives that were never part of the original trial, not an outcome associated with the original trial, the title should not have RCT mentioned. It is misleading

**Response:** We have revised the title to more clearly communicate this information.

Methods: Intervention description: The authors should clearly state and describe what the "tenth step" of the BFHI entailed? What was the frequency of the community support groups? Who led these? Were these peers based or supervised in any way? What was the content of these groups?

***Response:*** *We* *have provided additional description of the intervention implementation and refer the reader to the reports describing these in their full details.*

Control Condition description should be done. at the moment not enough details given

***Response****: We have provided additional description for the standard of care (lines 160-162).*

Recruitment: There seems to be a clear bias in recruitment - based on if the women wanted or not to be part of the BFHI. This is not random at all. The authors should comment on this.

***Response:*** *There is probably a misunderstanding. BFHI steps were implemented at the clinic/site level. To clarify we have added the eligibility criteria in the method: line 171-174.*

Analyses: Authors do not mention adjusting fro clustering, since this was a cRCT. A statistical review should be done to help authors in reporting. There seems to be some clear imbalances like domestic violence, education etc. Authors should state what was there approach, did they adjust for it? Also no mention about ITT analysis? If not ITT based analysis then why?

***Response:*** *All analyses are virtually ITT. Adherence data was not measure at the individual level and cross-over was simply treated as LTFU because we did not have the outcomes for participants who have moved out. The ITT results that rely on cluster randomization are reported as “Unadjusted” in Table 4 and “Mediation IPW” in Table 5, Figure 1, and Figure 2. The imbalances that occurred were accounted for using inverse probability of treatment weights. Furthermore, we accounted for potentially informative censoring with inverse probability weights as well. We state these approaches in the statistical analysis section of the Methods, provide several references for how inverse probability weights account for imbalances in that section, and provide a description of how this analytical method works in the Supplementary S2 file.*

*We note the difficulties adjusting for clustering in our response to reviewer 1.*

Discussion: Should report or mention what the results were of the original trial? Did BFHI improve early initiation and EBF rates. This will help set the context for the findings of this paper. Since one of the outcomes the authors report is the "meditational aspect of feeding issues". The discussion should highlighted the limitations eg loss to follow up, baseline imbalances or issues with recruitment bias etc

***Response:*** *We have reframed the discussion to discuss the limitations of the study. We note that we used several analytical approaches to weaken the assumptions regarding loss-to-follow-up and baseline imbalances.*

1.	Yotebieng M, Labbok M, Soeters HM, Chalachala JL, Lapika B, Vitta BS, et al. Ten Steps to Successful Breastfeeding programme to promote early initiation and exclusive breastfeeding in DR Congo: a cluster-randomised controlled trial. The Lancet Global health. 2015;3(9):e546-55. Epub 2015/08/08. doi: 10.1016/s2214-109x(15)00012-1. PubMed PMID: 26246225.
