# Supplementary material for: Postpartum depressive symptoms following implementation of the 10 steps to successful breastfeeding program in Kinshasa, Democratic Republic of Congo: A cohort study
Source: PLoS Med. 2021 Jan 11;18(1):e1003465. doi: 10.1371/journal.pmed.1003465 (PMC7799755; doi:10.1371/journal.pmed.1003465)
Supplement: Attachment — Submitted filename: BFHI_Revision 3 rebutal letter.docx [file pmed.1003465.s009.docx]

*We thank the editors for their feedback and consideration of our paper. Please see below for ours changes and responses.*

1. Response to academic editor's point 1: Thanks for revising the text in response to the academic editor’s comment regarding only considering one assessment of breastfeeding difficulties at 10 weeks.  However, we request that you please edit to clarify this text as follows:

"Similarly, only one assessment of breastfeeding difficulties (at week 10) was considered, even though this information was collected at each of the study visits (6, 10, 14, 18, and 24 weeks postpartum). In addition to the fact that earlier difficulties might be relatively more stressful, the increasing protective benefit of BFHI on depressive symptoms with increasing difficulties found in our mediation analysis suggest a potential cumulative effect of the number of difficulties overtime including a differential impact of transient vs persistent difficulties. The breastfeeding assessments at 6 and 10 weeks could have been incorporated into the mediation analysis by [please describe here the kind of analysis that could have been done with these additional assessments and how they would have added to the analysis described in the present manuscript]. Such relationships might fruitfully be explored in future analyses."

We have revised the text as requested.

2. Abstract: Methods and Findings:  Please provide some summary demographics on the women included in the study such as age, parity, exclusive breastfeeding status, and proportions reporting any breastfeeding difficulty.

We have added the requested information to the Abstract.

3. Abstract: Methods and Findings: Line 39-40: Please revise to: “However, a limitation of the study is that the results are based on two hospitals randomized to each group.”’

We have revised the text as requested.

4. Abstract: Conclusions: Line 43: Please change “resulting in” to “associated with” and at Line 44, please remove the word “huge”

We have revised the text as requested.

5. Author summary: What did the researchers do and find?: In the second bullet point, please change “resulting in” to “associated with”

We have revised the text as requested.

6. Author summary: What do these findings mean?:  Please summarize the findings of your study, such as “Our results suggest that implementation of the baby-friendly hospital initiative steps was associated with a reduction in depressive symptoms, and that breastfeeding difficulties play a role in this relationship.”

We have revised the text as requested.

7. Introduction: Line 127-128: Please revise to “Our objective was to quantify the association between implementing BFHI Steps 1-9 or Steps 1-10 and postpartum depressive symptoms.”

We have revised the text as requested.

8. Methods: Please specify the significance level used (eg, P<0.05, two-sided) and the statistical test used to derive a p value.

We have added a sentence clarifying that both the confidence limits and p-values come from a robust variance estimator, which provides conservative coverage (i.e. greater than 95% confidence limit coverage for confidence limits, rejects the null less than 5% of the time when the null hypothesis is true).

As we did not conduct null hypothesis significance testing, we did not have a significance level that was used. Not using a strict null hypothesis significance testing approach has been increasingly recommended (Szucs & Ioannidis 2017, McShane et al. 2019, Lash 2017) and is also in accordance with the American Statistical Association’s recent statement and guidance (Wasserstein & Lazar 2016). The American Statistical Association is one of the largest and oldest communities of statisticians. While what should replace the null hypothesis significance testing framework is still unclear (as indicated in recent publications in The American Statistician), we opted to use a estimation framework. The estimation framework has been increasingly used in epidemiology (Poole 2001, Stang et al. 2016, Rothman 1978, International Committee of Medical Journal Editors 1988, Gardener & Altman 1986).

Szucs, D., & Ioannidis, J. (2017). When null hypothesis significance testing is unsuitable for research: a reassessment. *Frontiers in human neuroscience*, *11*, 390.

McShane, B. B., Gal, D., Gelman, A., Robert, C., & Tackett, J. L. (2019). Abandon statistical significance. *The American Statistician*, *73*(sup1), 235-245.

Lash, T. L. (2017). The harm done to reproducibility by the culture of null hypothesis significance testing. *American journal of epidemiology*, *186*(6), 627-635.

Wasserstein, R. L., & Lazar, N. A. (2016). The ASA statement on p-values: context, process, and purpose.

Poole, C. (2001). Low P-values or narrow confidence intervals: which are more durable?. *Epidemiology*, *12*(3), 291-294.

Stang, A., Deckert, M., Poole, C., & Rothman, K. J. (2017). Statistical inference in abstracts of major medical and epidemiology journals 1975–2014: a systematic review. *European journal of epidemiology*, *32*(1), 21-29.

Rothman, K. J. (1978). A show of confidence.

International Committee of Medical Journal Editors. (1988). Uniform requirements for manuscripts submitted to biomedical journals. *British Medical Journal (Clinical research ed.)*, *296*(6619), 401.

Gardner, M. J., & Altman, D. G. (1986). Confidence intervals rather than P values: estimation rather than hypothesis testing. *Br Med J (Clin Res Ed)*, *292*(6522), 746-750.

9. Results: Lines 269-270: Please remove the word “substantively” from this sentence, and replace with “significantly” if statistical significance is meant (and please provide the 95% CIs/p values associated with the results here) because “substantively” is subjective (for example, 67% and 75% prevalence of difficulty may not be very different).

We have removed ‘substantively’. We do not intend ‘significantly’ because we did not rely on null hypothesis significance testing for interpretation of our results. A p-value less than 0.05 may indicate a difference between 67% and 68%, but be of little value.

As first described by Neyman and Pearson, significance cut-points for p-values are meant to be subjective determinations (“If we reject $H_{0}$, we may reject it when it is true; if we accept $H_{0}$, we may be accepting it when it is false, that is to say, when really some alternative $H$, is true. These two sources of error can rarely be eliminated completely; in some cases it will be more important to avoid the first, in others the second. … From the point of view of mathematical theory all that we can do is to show how the risk of the errors may be controlled and minimized. The use of these statistical tools in any given case, in determining just how the balance should be struck, must be left to the investigator.” Neyman & Pearson 1933). Therefore, statistical significance in regards to null hypothesis testing is inherently subjective as well, with subjectivity coming in with the choice of the cut-off. The selection of 0.05 is an arbitrary subjective tradition that masquerades as objectivity.

Neyman, J., & Pearson, E. S. (1933). IX. On the problem of the most efficient tests of statistical hypotheses. *Philosophical Transactions of the Royal Society of London. Series A, Containing Papers of a Mathematical or Physical Character*, *231*(694-706), 289-337.

10. Results: Lines 278-280: Please clarify this sentence to read “Additionally, the prevalence of any reported difficulties was observed to be higher for steps 1- 9, although this did not reach statistical significance (0.08; 95% CL: -0.01, 0.17; p=0.06), and was significantly higher steps 1-10 (0.22; 95% CL: 0.14, 0.30; p<0.01).”

Again, as we did not interpret our results based on null hypothesis statistical testing, the addition of this language is not how we interpreted the results. We have added the p-values as the editors previously requested, but we did not use them to interpret our results. With the p-values included readers are free to apply significance testing to the results if they desire. Use of p-values in interpretations as requested by the editors have led to incorrect claims in the past (see examples in Lash et al. 2018, Stang et al. 2010, *Modern Epidemiology* 3^rd^ Edition Chapter 10 Figure 10-5). As such, we would prefer that our work does not become a future example of this mistake.

Also as noted in the methods, the robust variance estimator provides conservative estimation of the variance. Therefore, the reported p-values are based on a variance larger than the true variance. Therefore, the editors are over-interpreting a p-value that is expected to be larger than true. In the estimation framework, the conservative variance is less problematic compared to the null hypothesis significance testing for results near alpha.

Lash, T. L., Collin, L. J., & Van Dyke, M. E. (2018). The replication crisis in epidemiology: snowball, snow job, or winter solstice?. *Current Epidemiology Reports*, *5*(2), 175-183.

Stang, A., Poole, C., & Kuss, O. (2010). The ongoing tyranny of statistical significance testing in biomedical research. *European journal of epidemiology*, *25*(4), 225-230.

Rothman, K. J., Greenland, S., & Lash, T. L. (Eds.). (2008). *Modern epidemiology*. Lippincott Williams & Wilkins.

11. Results: Lines 287-289: Please revise this sentence to avoid confusion (it isn’t clear what a minor increasing association is), and please present the point estimate with 95%CIs and p values in the text: “When no breastfeeding difficulties occurred, we did not find evidence of a significant association of either intervention with EPDS scores or probable depression (Table 5)”

We have revised the text as: “When no breastfeeding difficulties occurred, there was a positive but imprecise association of both interventions with EPDS scores or probable depression (Table 5).”

12. Results: Lines 289-291: In line with the above revision, the following two sentences can be removed: “However, these estimates are small and imprecise, as indicated by the width of the confidence intervals. Therefore, there is insufficient indication of increased EPDS scores for either intervention if no breastfeeding difficulties was held constant.”

We have revised the text. However, we chose to keep the second sentence for emphasis purposes.

13. Results: Lines 322-330: Please revise these sentences to reflect the fact that there were no significant increases in EPDS or probable depression with the zero reported difficulties breastfeeding, and as shown in table 3.1, there are also no significant changes in EPDS or probably depression under zero reported difficulties.  “The controlled direct association for both EPDS scores and probable depression reversed directions under no breastfeeding difficulties (Supplemental Table 3.1). However, similar to the results for mediation for Steps 1-9 presented in Table 5, changes in EPDS scores and probable depression under no difficulties breastfeeding did not reach statistical significance, suggesting that there is no association between steps 1-9 and EPDS at no breastfeeding difficulties at week 10.”

Again, we did not use a null hypothesis significance testing framework for the reasons stated previously. We have revised our language to reflect that our observed results are compatible with both a null association or a weak association.

14. Discussion: Lines 339-340: Please revise this to “...with no evidence of an association of the BFHI steps with depressive symptoms in the absence of breastfeeding difficulties.”

We have revised the text as requested.

15. Discussion: Lines 366-369: Please revise this to reduce causal language: “Our findings that the BFHI was associated with reduced depressive symptoms…”

We have revised the text as requested.

16. Discussion: Lines 433-435: This sentence is confusing as there was no significant effect identified, please remove or clarify to: “As indicated by our analyses, we did not identify a significant controlled direct association for no breastfeeding difficulties for steps 1-9.”

As previously stated, we did not use null hypothesis significance testing. If readers wish to interpret the results as the editors do, then they have the p-values necessary to do so. However as indicated in the previously referenced example, this can lead to incorrect interpretations and is not objective.

17. Conclusions: Lines 439-440: Please revise to “In conclusion, we have shown that implementation of the BFHI steps is associated with a decrease in depression symptoms at 14-weeks postpartum.”

We have revised the text as requested.

18. Table 2 and Table 3: Please make it clear in the legend or the Table headings that “Steps 1-9” and “Steps 1-10” refer to the BFHI (and please spell out the abbreviation).  In Table 2, the comparison group is “Standard of Care” and in Table 3 this is “Control” - please use consistent terminology throughout.

We have updated the table footnotes to make it clear that the steps refer to BFHI. Additionally, we have updated Table 3 to ‘Standard of Care’.

19. S5 Data file: Please provide a summary tab, or similar to explain the variable names and cell values.

We have included an additional tab in the data file as requested.
